# Microscopic Insights into Cation-Coupled Electron Hopping Transport in a Metal–Organic Framework

**DOI:** 10.1021/jacs.1c13377

**Published:** 2022-03-24

**Authors:** Ashleigh T. Castner, Hao Su, Erik Svensson Grape, A. Ken Inge, Ben A. Johnson, Mårten S. G. Ahlquist, Sascha Ott

**Affiliations:** † Department of Chemistry−Ångström Laboratory, Uppsala University, Box 523, 75120 Uppsala, Sweden; ‡ Department of Theoretical Chemistry and Biology, KTH Royal Institute of Technology, 10691 Stockholm, Sweden; § Department of Materials and Environmental Chemistry, Stockholm University, 106 91 Stockholm, Sweden

## Abstract

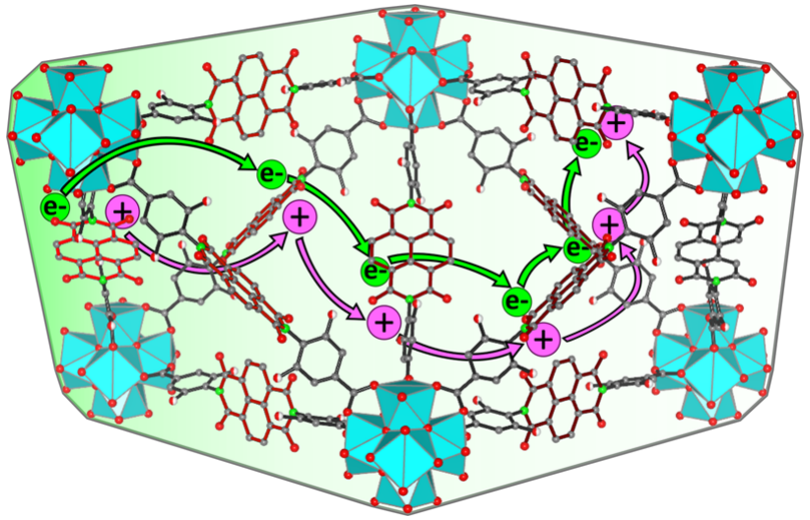

Electron transport through metal–organic frameworks by a hopping mechanism between discrete redox active sites is coupled to diffusion-migration of charge-balancing counter cations. Experimentally determined apparent diffusion coefficients, *D*_*e*_^app^, that characterize this form of charge transport thus contain contributions from both processes. While this is well established for MOFs, microscopic descriptions of this process are largely lacking. Herein, we systematically lay out different scenarios for cation-coupled electron transfer processes that are at the heart of charge diffusion through MOFs. Through systematic variations of solvents and electrolyte cations, it is shown that the *D*_*e*_^app^ for charge migration through a PIZOF-type MOF, Zr(dcphOH-NDI) that is composed of redox-active naphthalenediimide (NDI) linkers, spans over 2 orders of magnitude. More importantly, however, the microscopic mechanisms for cation-coupled electron propagation are contingent on differing factors depending on the size of the cation and its propensity to engage in ion pairs with reduced linkers, either non-specifically or in defined structural arrangements. Based on computations and in agreement with experimental results, we show that ion pairing generally has an adverse effect on cation transport, thereby slowing down charge transport. In Zr(dcphOH-NDI), however, specific cation–linker interactions can open pathways for concerted cation-coupled electron transfer processes that can outcompete limitations from reduced cation flux.

## Introduction

Metal–organic frameworks (MOFs) are a class of porous crystalline materials, which are composed of inorganic nodes, often in the form of secondary binding units (SBUs), and polydentate organic linkers.^1,2^ These components self-assemble to form periodic constructs with a long-range order that exhibits both high internal surface areas and permanent porosity.^3−5^ Due to their modular nature, MOFs can be constructed from a vast array of different SBUs and linkers, enabling the preparation of functional MOF materials for a variety of applications,^6−10^ notably the molecular catalysis of electrochemical reactions.^11−13^

Because MOFs are often constructed from redox-inert linkers and metal clusters, they are generally insulating in nature. However, a substantial amount of recent work has focused on preparing electroactive MOF materials for electrochemical applications where an electrode is modified with a MOF film or particles.^14,15^ Electron transfer at the electrode–film interface as well as in the bulk of the MOF material takes place in the presence of solvent molecules and a supporting electrolyte composed of redox-inert ionic species. In these reports, charge transport is generally established by one of two macroscopic mechanisms. The first of which is conventional ohmic conduction that relies on sizable orbital overlap and electronic coupling between the molecular components of the framework, either with through-bond or through-space approaches.^16^ Alternatively, electron transport may take place in an outer-sphere manner, in which charges propagate via redox-hopping between isolated electroactive components. Many of the recently reported electroactive MOFs employ the latter strategy, and electroactive building blocks include redox-active organic linkers,^17−21^ metallo-linkers,^22−28^ and open metal sites (e.g., Cu ions^29^ or clusters^30^). While such redox-active MOFs that rely on charge hopping transport have garnered much attention in recent years for potential applications like energy storage and electrocatalysis, microscopic details on charge transport by the electron hopping mechanism are still unclear, and little is understood about the influence of all contributing factors.

It is generally accepted that charge transport in MOFs is influenced by mass transfer of a requisite counter ion, which maintains charge neutrality and promotes further redox hopping.^22,31−33^ This charge transport process can be affected by a number of factors including the following: (1) MOF pore size and size of the charge-balancing counter ion, both of which affect the physical capability of the counter ion to be transported to the interior of the MOF, (2) strength of ion pairing, which would influence the rate of counter ion ingress into the MOF, and (3) the concentration of available counter ions to balance charges of reduced/oxidized species in the MOF. These elements collectively contribute to the mass transport of the charge-balancing counter ion into the MOF to support linker-to-linker electron hopping with diffusion-like behavior.

Ion transport has been explored and discussed in various electroactive MOF materials to assess some parameters like pore-size,^34^ concentration of counter ions,^19^ and electrolyte in general.^35^ The majority of these studies have focused on the influence of the size of either the MOF architecture or the counter ion. To the best of our knowledge, no systematic studies on the composition of the electrolyte medium, particularly the influence of the solvent, have been explored for assessing charge transport in redox-active MOFs, even though it has been noted as a potential influencing factor.^36,37^

**Figure 1:**
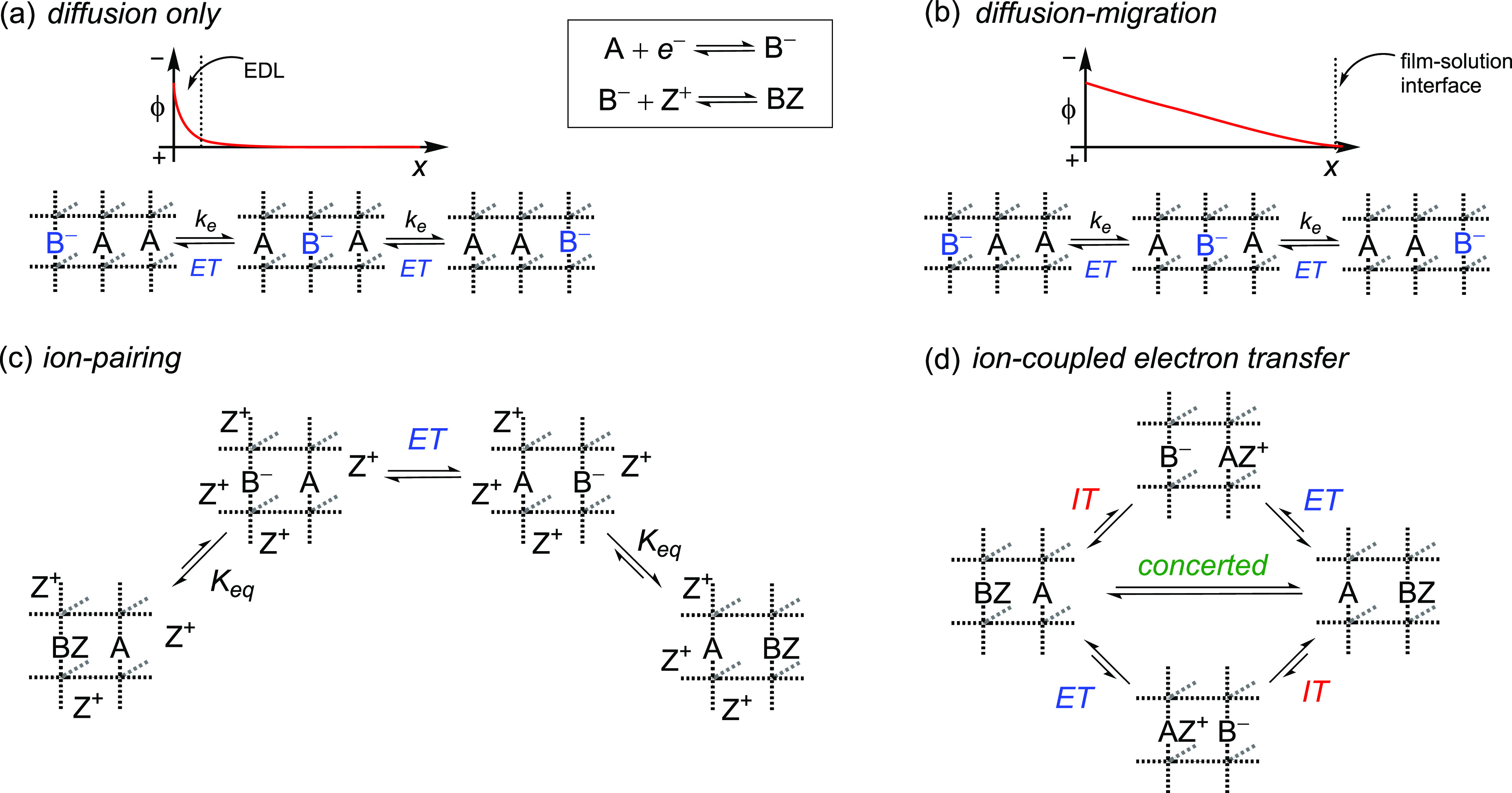
Schematic diagrams showing various microscopic mechanisms of electron-hopping through a redox-active MOF film: (a) Diffusion in the absence of any other effects, fulfilling assumptions made in eq 1; the electrostatic potential (ϕ) is dropped only over the electrical double layer (EDL) near the electrode surface and no electric field is developed within the film. (b) Charge transport by diffusion-migration, where there exists a substantial drop in electrostatic potential across the film. (c) Ion pairing electron transfer where the microscopic reactions include dissociation/association of an ion pair as well as electron self-exchange between an unpaired reduced/oxidized linker. These reactions are accompanied by migration-diffusion of redox-inactive counter ions according to the Nernst–Planck equation under an electroneutrality assumption. (d) Ion-coupled electron transfer occurring from fully associated ion-paired linkers. The microscopic self-exchange reaction follows an ion-coupled electron transfer (ICET) process, which can be represented by a square scheme showing either sequential or concerted pathways.

The interpretation of the charge transport properties of a redox-active MOF (e.g., the transient current response after a large potential step) will ultimately depend on the microscopic mechanism.^38^ In the simplest model, many individual electron self-exchange reactions take place at the molecular scale between adjacent layers of discrete redox-active linkers (Figure 1). When considered globally, the net movement of charge as a result of these self-exchange reactions exhibits diffusion-like behavior. The diffusion coefficient (*D_e_*) is then related to the rate of the self-exchange reaction^39^ by
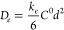
1where *k_e_* (M^–1^ s^–1^) is the second order self-exchange rate constant, *C*^0^ is the total concentration of redox-active molecules, and *d* is the average hopping distance.

This treatment assumes that a supporting electrolyte permeates the pores of the MOF, such that there is no electric field in the bulk of the film (Figure 1a). Additionally, strong intermolecular interactions, either between the linkers themselves or ion-pairing between the linker and the redox-inactive counter ions, are assumed to be absent.

Experimentally, an apparent macroscopic diffusion coefficient, which we will denote as *D*_*e*_^app^, can be obtained from chronoamperometry by measuring the transient current after applying a large potential step to the MOF film electrode.^15,40^ Macroscopically, these measurements essentially take the form of Fick’s law: (1) apply a macroscopic gradient across the film and (2) measure the flux that arises in response to this perturbation. The proportionality constant between the macroscopic gradient and the measured flux will be *D*_*e*_^app^. In all the cases described herein, the early time current decay will be proportional to *t*^–1/2^, from which *D*_*e*_^app^ can be calculated (*vide infra*).^41,42^ If the assumptions described above are fulfilled under the experimental conditions, this macroscopically determined *D*_*e*_^app^ will be identical to the theoretical microscopic *D_e_* from eq 1, which reflects the kinetics of the self-exchange reaction between linkers.

While this provides a simple model relating a microscopic mechanism to macroscopic charge transport, it is not sufficient to explain the dependence of diffusion coefficients measured in this manner (*D*_*e*_^app^) on the nature of the counter ion, as is often observed for many MOF films.^17,26,34,35^ Accordingly, a refinement of the above model needs to include the coupling of electron hopping between localized sites and transport of electro-inactive counter ions through the framework. In an earlier report, including electromigration of the mobile counter ion under the constraint of electroneutrality (Figure 1b) was used to explain deviations from eq 1 observed in redox-polymer modified electrodes.^42^ This physico-mathematical model quantitatively predicts that restricted mobility of the counter ions leads to an electric field (electrostatic potential drop across the film) that enhances electron-hopping transport over the case of purely diffusion. In other words, the effect of migration grows as the diffusivity of the counter ion (*D*_I_) decreases, resulting in larger current responses and leading to a large overestimation of *D*_*e*_^app^ (). For example, fitting of the data including migration effects from an Os/Ru copolymer film resulted in *D*_*e*_^app^/*D_e_* = 5.5.^42^ The counter ion diffusion coefficient within the film was estimated to be much lower than that for electron-hopping transport, *D*_I_/*D_e_*≤ 10^–2^. This led to the conclusion that measured apparent diffusion coefficients do not necessarily represent the slower of the two concurrent processes: electron hopping and counter ion transport to maintain electroneutrality.

Further refinement to this picture includes the possibility of strong ion pairing interactions between the fixed redox-active molecule and the mobile counter ion.^41,43^ In this situation, there are several conceivable microscopic mechanistic pathways, displayed in Figure 1c,d. In the first scenario (Figure 1c), the ion pair in the preceding layer of the film must fully dissociate before the electron transfer takes place. This is accompanied by migration-diffusion of the dissociated counter ion. It was predicted that for this mechanism, the current response decreases as the ion pairing equilibrium constant increases.^43^ In other words, both the observed current and *D*_*e*_^app^ are decreasing functions of *K*_eq_ = *k*_A_/*k*_D_, the ion-pairing association equilibrium constant (Figure 1c).

Alternatively, in highly non-polar environments, it is possible that the electron transfer involves fully associated ion-paired linkers, resulting in the square scheme displayed in Figure 1d. In this case, the overall electron-ion exchange between consecutive layers in the film can occur in a sequential or concerted manner.^44−46^ The latter may provide a significant thermodynamic advantage as it avoids high-energy intermediates.

Herein, we present experimental and computational observations that suggest specific mechanisms for cation-coupled electron hopping transport. We will attempt to rationalize macroscopic observations of charge transport using the microscopic models outlined in Figure 1.

The MOF that was used for this study is a porous interpenetrated Zr-organic framework (PIZOF), *i.e.* a class of MOF with identical Zr_6_O_4_(OH)_4_ SBUs and 12-fold connectivity as the UiO-series of MOFs, but that is composed of two independent interpenetrating UiO networks.^47^ The MOF, hereafter termed Zr(dcphOH-NDI),^17^ is based on naphthalenediimide (NDI) linkers that are particularly useful for this study as they have been shown to engage in ion pairing with supporting electrolyte upon electrochemical reduction both in homogenous solutions^48^ and as monomers of heterogeneous polymer thin films.^49^ In the presence of non-coordinating electrolyte and/or highly polar solvents, the cyclic voltammogram (CV) of the NDI core is characterized by two sequentially ordered one-electron reductions.

Zr(dcphOH-NDI) was prepared as thin films solvothermally grown on conductive fluorine-doped tin oxide (FTO) substrates and employed as working electrodes in a series of chronoamperometry experiments to determine macroscopic apparent diffusion coefficients *D*_*e*_^app^. With a systematic variation of the counter ions (Li^+^, K^+^, and *n*-tetrabutylammonium (TBA^+^)) as well as the solvent (DMF, THF, and ethanol (EtOH)), it can be expected that different mechanistic regimes for cation-coupled electron hopping transport according to Figure 1 can be explored. It is demonstrated that the measured *D*_*e*_^app^ is significantly affected by various factors. Computational data are presented and discussed to explain some of the differences observed in the experimental outcomes. The results described herein shine light on the importance of the electrolyte composition for assessing electroactive MOF materials and the influence of this imposed experimental condition on the microscopic mechanism for electron hopping charge transport.

## Materials/Methods

### Synthesis and Characterization

**Figure 2:**
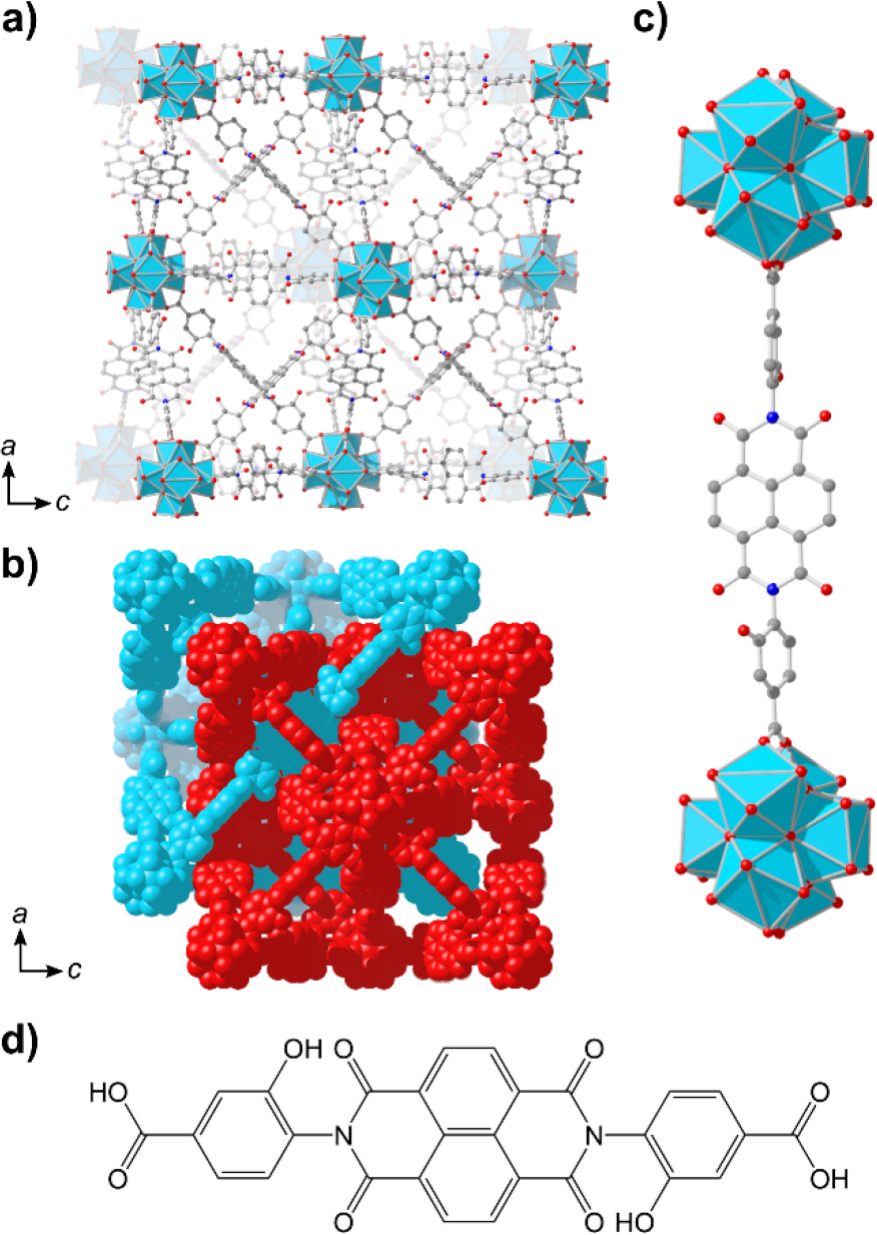
Structure of Zr(dcphOH-NDI) obtained by three-dimensional electron diffraction (3DED) measurements: (a) Non-interpenetrated framework, showing the hexanuclear zirconium clusters, as viewed slightly off-axis along *b*. (b) Two interpenetrated frameworks, colored blue and red. (c) Two hexanuclear zirconium clusters interconnected by a single dcphOH-NDI linker, showing the staggered confirmation of the NDI. (d) Chemical structure of the dcphOH-NDI linker.

The redox-active NDI linker dcphOH-NDI (see Figure 2d) was prepared as previously described.^17^ MOF thin films on FTO substrates from this linker were prepared by solvothermal synthesis in DMF with AcOH as a modulator at 120 °C for 72 h, as previously described.^17^ The resulting thin films, Zr(dcphOH-NDI)@FTO, were similar to those reported previously, exhibiting an interpenetrated PIZOF topology (see the Supporting Information for details, Figure S1). It is worthwhile noting that in contrast to the earlier report, the as-prepared films were not evacuated prior to voltammetric measurements. Non-evacuated Zr(dcphOH-NDI)@FTO films do not generally require an extensive conditioning period to exhibit maximum current densities (see the Supporting Information for details in Figure S1, experimental data in Figures S2–S7).

Crystals for structural characterization of the PIZOF Zr(dcphOH-NDI) were obtained from solvothermal synthesis, yielding micrometer-sized crystals that were used for three-dimensional electron diffraction (3DED) measurements (see the Supporting Information for details, Figure S8), allowing the collection of single-crystal diffraction data from micrometer- to nanometer-sized crystals.^50,51^ The structure of Zr(dcphOH-NDI) consists of two interpenetrated frameworks having a 12-c **fcu** net (Figure 2a,b), where each framework is made up by the archetypal hexanuclear zirconium SBUs, which are interconnected by dcphOH-NDI linkers. The resulting structure exhibits a maximum pore diameter of about 11 Å. Additionally, the structural investigation showed that the NDI moiety is staggered with respect to the carboxylate-bearing groups of the dcphOH-NDI linker (Figure 2c).

## Results/Discussion

### Electrochemical Analysis: CVs of the Homogeneous dcphOH-NDI Linker

Prior to the study of the Zr(dcphOH-NDI)@FTO films, the CVs of the homogeneous linker dcphOH-NDI in the solvent/supporting electrolyte systems of relevance were evaluated. It has previously been demonstrated that shifts in the observed potentials of the NDI^0/·–^ and NDI^·–/2–^ couples occur as a function of solvent polarity and the presence of Lewis acids such as Li^+^ or Mg^2+^ cations.^48^ These shifts are more pronounced for the NDI^·–/2–^ couple than for the NDI^0/·–^ couple, and their magnitude is strongly influenced by solvent properties like the dielectric constant (ε_r_) and donor ability.

**Figure 3:**
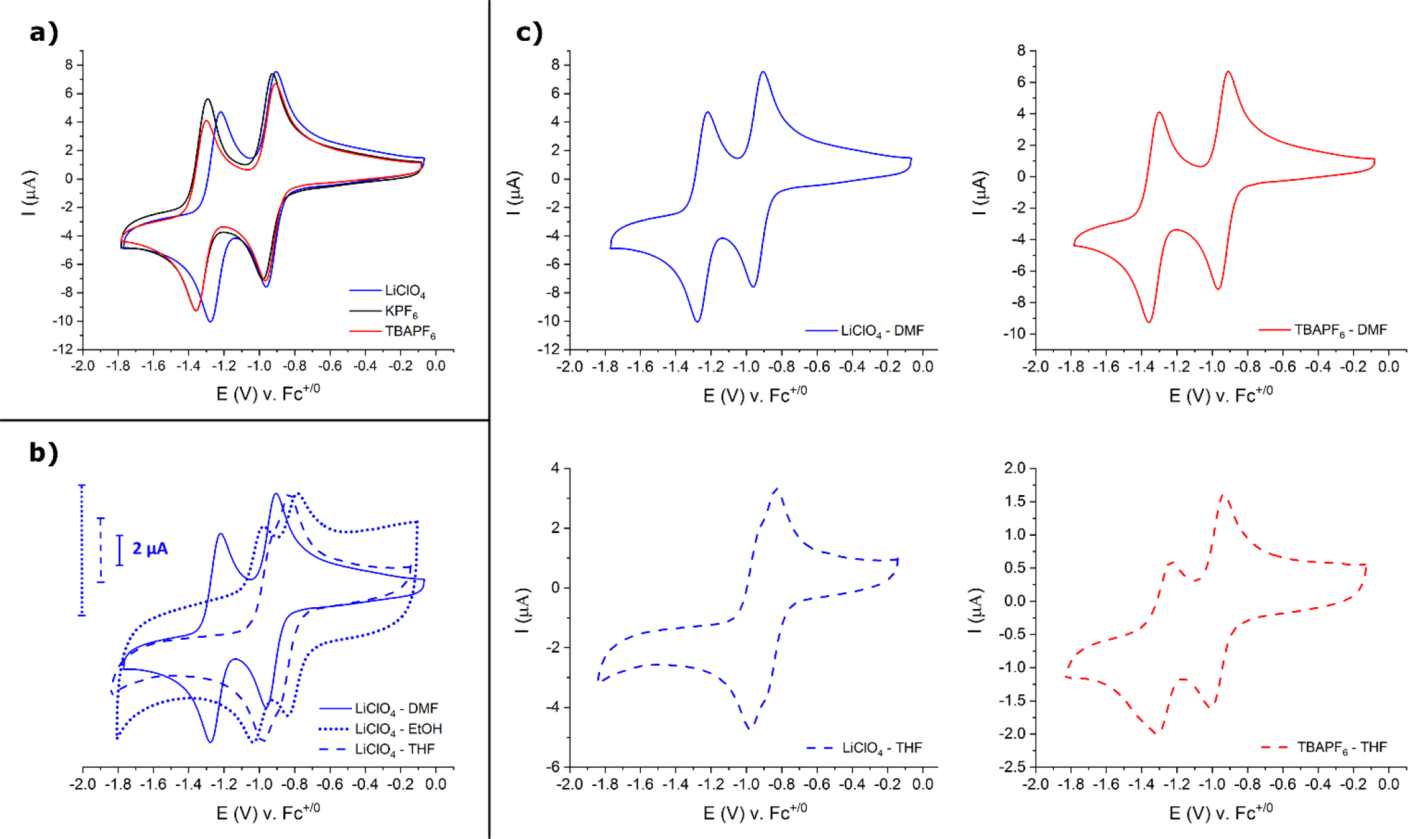
CVs of dcphOH-NDI measured under all supporting electrolyte/solvent conditions used in this study (0.5 M supporting electrolyte in the indicated solvent; ν = 50 mV s^–1^). (a) CVs measured in DMF with all supporting electrolytes tested. [dcphOH-NDI] = 1 mM for all measurements. (b) Normalized CVs measured in all solvents tested with LiClO_4_ as the supporting electrolyte. [dcphOH-NDI] = 1 mM in DMF (solid line) but is less than 1 mM in THF (dashed line) and EtOH (dotted line) due to low solubility of dcphOH-NDI in these solvents. Scale bars indicate actual measured current in each solvent, with the line style of the scale bar corresponding to that of the measured CV (refer to legend). (c) CVs illustrating alteration in NDI redox behavior resulting from the choice of supporting electrolyte (LiClO_4_: blue, TBAPF_6_: red) and solvent (DMF: solid lines, THF: dashed lines).

Accordingly, the CVs of dcphOH-NDI in Li^+^-, K^+^-, and TBA^+^-containing electrolytes (*r*_Li+_ = 0.76 Å,^52^*r*_K+_ = 1.33 Å,^53^ and *r*_TBA+_ = 4.94 Å^54^) in DMF solution do not differ dramatically, but they still exhibit subtle differences. While the *E*_1/2_ of the NDI^0/·–^ couple is essentially the same for all electrolytes, that of the NDI^·–/2–^ couple in LiClO_4_ is shifted positively by 80 mV compared to those in KPF_6_ and TBAPF_6_ (Figure 3a and Table 1). This shift is consistent with a fast chemical reaction subsequent to electron transfer, *i.e*., the formation of an ion pair between NDI^2–^ and the Li^+^ cation, that proceeds even in highly polar DMF solution.

**Table 1:** *E*_1/2_ for NDI Redox Couples of the Homogeneous Linker dcphOH-NDI^a^

	DMF		EtOH		THF	
supporting electrolyte	*E*_1/2_^0/·–^ (V)	*E*_1/2_^·–/2–^ (V)	*E*_1/2_^0/·–^ (V)	*E*_1/2_^·–/2–^ (V)	*E*_1/2_^0/·–^ (V)	*E*_1/2_^·–/2–^ (V)
LiClO_4_	–0.93	–1.25	–0.81	–1.00	–0.86	–0.95
KPF_6_	–0.95	–1.33				
TBAPF_6_	–0.94	–1.33			–0.97	–1.27

a Conditions: 0.5 M supporting electrolyte in indicated solvent; scan rate: 50 mV s^–1^; potentials referenced to Fc^+/0^.

The effect of ion pairing becomes more pronounced when the solvent is changed from DMF (ε_r_ = 36.7) to a solvent with a lower dielectric constant, like EtOH (ε_r_ = 24.3) or THF (ε_r_ = 7.5). As shown in Figure 3b, the CVs of dcphOH-NDI in the three solvents using LiClO_4_ as the supporting electrolyte differ significantly. It is apparent that the observed *E*_1/2_ of the NDI^0/·–^ couple is positively shifted by 120 and 70 mV for EtOH and THF, respectively, compared to that in DMF. Contributing factors that lead to this shift include most likely different degrees of stabilization of the NDI radical anion by the different solvents, as well as fast cation association after reduction. Stronger ion pairing can be expected for the dianion, as indicated by the potential of the NDI^·–/2–^ couples, which are shifted positively by 250 and 300 mV when going from DMF to EtOH and THF, respectively.

The *E*_1/2_ values of the NDI^0/·–^ and NDI^·–/2–^ redox couples in TBA^+^-containing electrolyte are basically insensitive to changes in solvent composition (see Figure 3c and Table 1, TBA^+^ in EtOH is not shown due to low solubility of TBAPF_6_), suggesting that TBA^+^ does not engage in sizable association with the reduced NDI species. Thus, the *E*_1/2_ values that are measured in TBA^+^ electrolyte can be regarded as reference potentials against which potentials that were obtained with other electrolytes can be compared to. The difference between the *E*_1/2_ of the NDI^·–/2–^ couple for Li^+^- as compared to TBA^+^-containing electrolyte is 80 mV in DMF, while it amounts to 320 mV in THF. These shifts are indicative of increasingly strong Li^+^ association when going to increasingly less polar solvents.

These observations for the homogenous linker allow predictions for the behavior of the MOF thin films. In low dielectric solvents and with high charge density cations, it is more likely that electron propagation through the film occurs via an ion paired state, as outlined in Figure 1c,d. In the opposite case, *i.e.*, in polar solvents and low charge density cations, ion pairing interactions are weaker, and the cations may be expected to be more freely diffusing. This scenario would favor electron transport through the MOF from a cation dissociated state, as depicted in Figure 1b. However, in the confinement of the MOF, diffusion-migration of large cations such as TBA^+^ may be limited by the physical size of the ion more than any other phenomena, presenting an additional factor that can affect *D*_*e*_^app^.

### Electrochemical Analysis: CVs of Zr(dcphOH-NDI)@FTO Films

**Figure 4:**
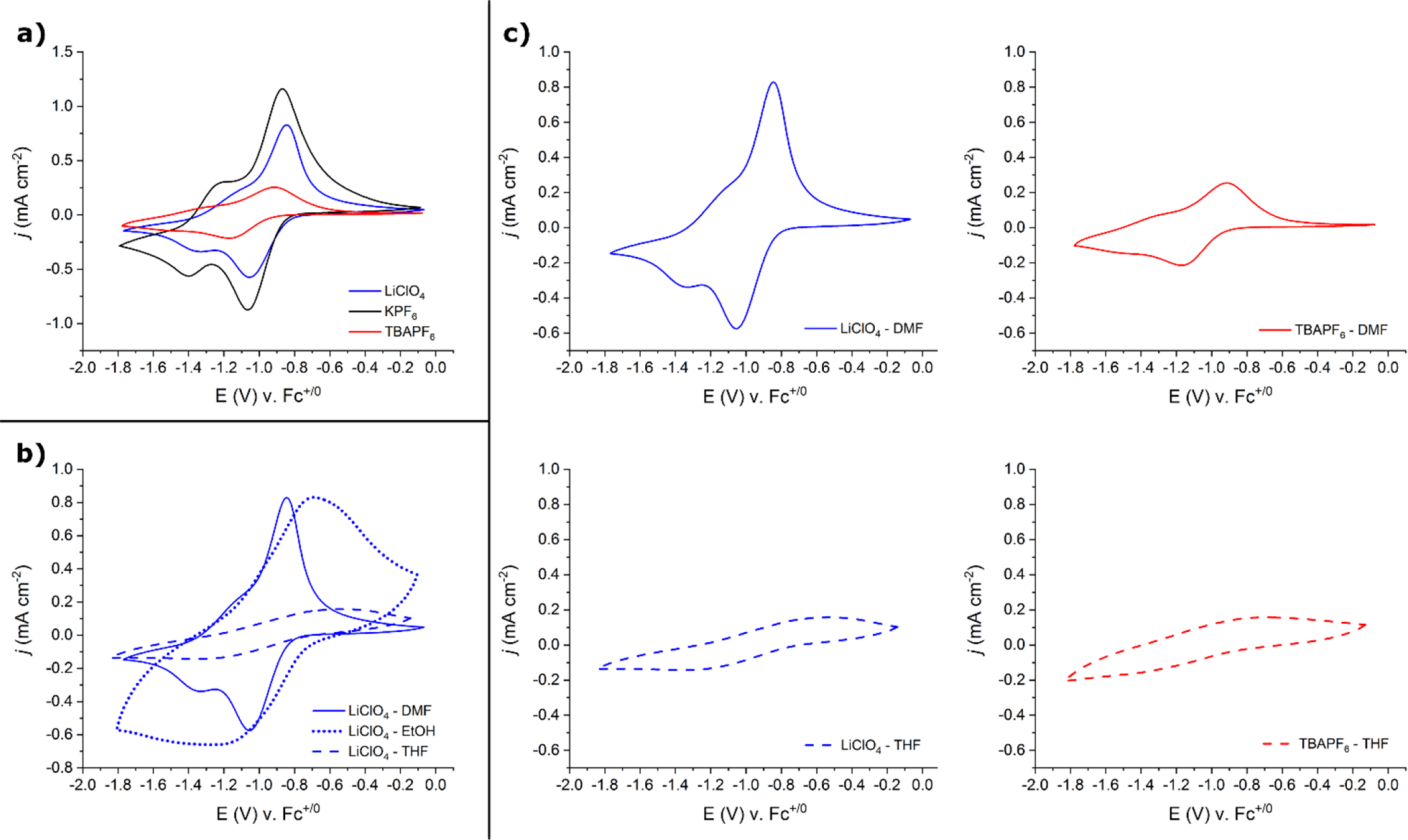
Representative CVs of Zr(dcphOH-NDI)@FTO MOF films after conditioning measured under all supporting electrolyte/solvent conditions used in this study. (a) CVs measured in DMF with all supporting electrolytes tested. (b) CVs measured in all solvents tested with LiClO_4_ as the supporting electrolyte. (c) CVs demonstrating the change of Zr(dcphOH-NDI)@FTO redox behavior stemming from the choice of supporting electrolyte (LiClO_4_: blue, TBAPF_6_: red) and solvent (DMF: solid lines, THF: dashed lines). CV conditions: 0.5 M supporting electrolyte in the indicated solvent; scan rate: 50 mV s^–1^.

Representative CVs of the Zr(dcphOH-NDI)@FTO films in the relevant electrolyte/solvent systems are shown in Figure 4 and summarized in Table 2. First, focusing on the CVs in DMF in Figure 4a, distinct differences can be observed depending on the nature of the electrolyte. For the smaller Li^+^ and K^+^ cations, the two redox features associated with the NDI^0/·–^ and NDI^·–/2–^ couples are well defined and characterized by higher current densities than those recorded with the bulkier TBA^+^ as supporting electrolyte. The *E*_1/2_ of the NDI^·–/2–^ couple measured with Li^+^ and K^+^ is positively shifted with respect to that measured with TBA^+^ (130 and 40 mV for Li^+^ and K^+^, respectively, see Table 2). Following the same reasoning as above, Li^+^ cations form stronger ion pairs with the NDI^2–^ state in the MOF film as compared to K^+^. Such effects can be expected to be considerably less significant for CVs in the bulky TBA^+^ electrolyte. That being the case, the sheer size of the TBA^+^ cation slows down ion diffusion-migration, leading to CVs with significantly lower current densities. In the absence of sizeable ion pairing, the *E*_1/2_ of the NDI^0/·–^ couple in the TBA^+^ electrolyte is observed at 90 and 70 mV more negative potentials than that with Li^+^ and K^+^ electrolytes, respectively.

**Table 2:** *E*_1/2_ for NDI Redox Couples of Zr(dcphOH-NDI)@FTO in DMF^a^

	DMF	
supporting electrolyte	E_1/2_^0/•–^ (V)	E_1/2_^•–/2–^ (V)
LiClO_4_	–0.95	–1.21
KPF_6_	–0.97	–1.30
TBAPF_6_	–1.04	–1.34

a Conditions: 0.5 M electrolyte in DMF; scan rate: 50 mV s^–1^; potentials referenced to Fc^+/0^.

Keeping the Li^+^ electrolyte constant, but changing the solvent from DMF to either EtOH or THF (Figure 4b), two main effects become apparent in the CVs. First, the peak-to-peak separations in the CVs increase to such an extent that the NDI^0/·–^ and NDI^·–/2–^ redox couples are no longer discernable. Large peak-to-peak separations are not uncommon in MOF electrochemistry and are usually attributed to sluggish interfacial electron transfer, ohmic resistance in the film, and/or slow ion transport at the film|electrolyte interface. Second, the current densities of CVs in THF are significantly lower than those in DMF or EtOH. In fact, the current densities in THF are similar irrespective of whether Li^+^ or TBA^+^ are used as electrolytes (Figure 4c). While the reasons for this phenomenon are most likely different, *i.e*. strong ion pairing for the former and sterically hindered cation diffusion-migration for the latter electrolyte, the low current densities suggest that apparent diffusion coefficients would be lower in THF as compared to DMF.

Summarizing the discussion of the CVs of the Zr(dcphOH-NDI)@FTO films, the current responses for a given charge-balancing cation in the MOF film are clearly solvent dependent. Increased ion pairing results in a lower ion transport in the MOF film, which in turn would favor mechanisms for electron propagation through the film which start from cation-associated states, as depicted in Figure 1c,d. Furthermore, by selecting a solvent that promotes stronger ion-pairing between the cation and reduced NDI, the mode for redox-hopping charge transport through the MOF film can be expected to be more ion-coupled rather than being only influenced by ion size.

### Chronoamperometry and Cottrell Analysis for *D*_*e*_^*app*^ Determination: Zr(dcphOH-NDI)@FTO

The apparent diffusion coefficients *D*_*e*_^app^ for charge diffusion through Zr(dcphOH-NDI)@FTO films to produce the NDI radical anion state in the various solvents were determined by chronoamperometry. These experiments were performed by first applying a potential in the non-faradaic region positive of the first wave for 90 s to ensure all NDI linkers are in a neutral state before stepping the potential to a sufficiently more negative value to reduce the linkers to the radical anion. The potential steps chosen for the analyses were determined from CVs (samples in DMF) or from differential pulse voltammetry (other solvents). The recorded time-dependent current responses were plotted to calculate the concentration of electroactive NDI (Γ_*e*_) on the surface from the following relationship:
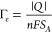
2where *Q* is the charge passed (in C) after exhaustive reduction of the film, *n* is the number of electrons transferred, *F* is Faraday’s constant, and *S_A_* (in cm^2^) is the geometric surface area of the Zr(dcphOH-NDI)@FTO electrode (see the Supporting Information for details). As summarized in Table 3, the surface concentration of electroactive NDI (Γ_*e*_) is very similar for all electrolyte/solvent combinations, thereby also demonstrating low film-to-film variations between the analyzed Zr(dcphOH-NDI)@FTO electrodes. All variables for determining Γ_*e*_ and *D*_*e*_^app^ reported herein were measured independently for three samples assessed in each electrolyte/solvent mixture to ensure accuracy of the reported values (see the Supporting Information for details).

**Table 3:** Average Γ_*e*_ and *D*_*e*_^app^ Measured for the NDI^0/·–^ Redox Couple for Zr(dcphOH-NDI)@FTO MOF Films (Standard Deviations Were Obtained from Three Samples Measured Independently for All Entries)

	DMF		EtOH		THF	
supporting electrolyte	Γ_*e*_ (mol cm^–2^)	*D*_*e*_^app^ (cm^2^ s^–1^)	Γ_*e*_ (mol cm^–2^)	*D*_*e*_^app^ (cm^2^ s^–1^)	Γ_*e*_ (mol cm^–2^)	*D*_*e*_^app^ (cm^2^ s^–1^)
LiClO_4_	5.99 ± 0.39 × 10^–8^	3.23 ± 2.16 × 10^–9^	5.95 ± 0.93 × 10^–8^	2.36 ± 1.55 × 10^–10^	6.82 ± 1.27 × 10^–8^	1.30 ± 0.24 × 10^–11^
KPF_6_	7.92 ± 1.91 × 10^–8^	1.12 ± 0.30 × 10^–9^				
TBAPF_6_	3.93 ± 1.77 × 10^–8^	6.99 ± 5.97 × 10^–10^			3.96 ± 2.82 × 10^–8^	2.80 ± 1.04 × 10^–11^

With the surface concentration of electroactive NDI linkers in hand, the apparent diffusion coefficient (*D*_*e*_^app^) for charge diffusion can be extracted from the Cottrell relationship:
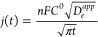
3where *j*(*t*) is the time-dependent current density (in A cm^–2^) and *C*^0^ is the molar concentration of electroactive NDI species (in mol cm^–3^). *C*^0^ is defined as the electroactive surface concentration (Γ_*e*_) divided by the film thickness (*d_f_*) (in cm) as obtained from cross-section SEM (see the Supporting Information for details). For sufficiently short time transients, it is expected that charge diffusion within the film will be in a semi-infinite regime. For the timeframe where this condition holds, the Cottrell plot of *j*(*t*) *vs t*^–1/2^ will be linear such that the slope of the plot can be used to extract the *D*_*e*_^app^ from the following expression:
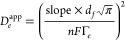
4

**Figure 5:**
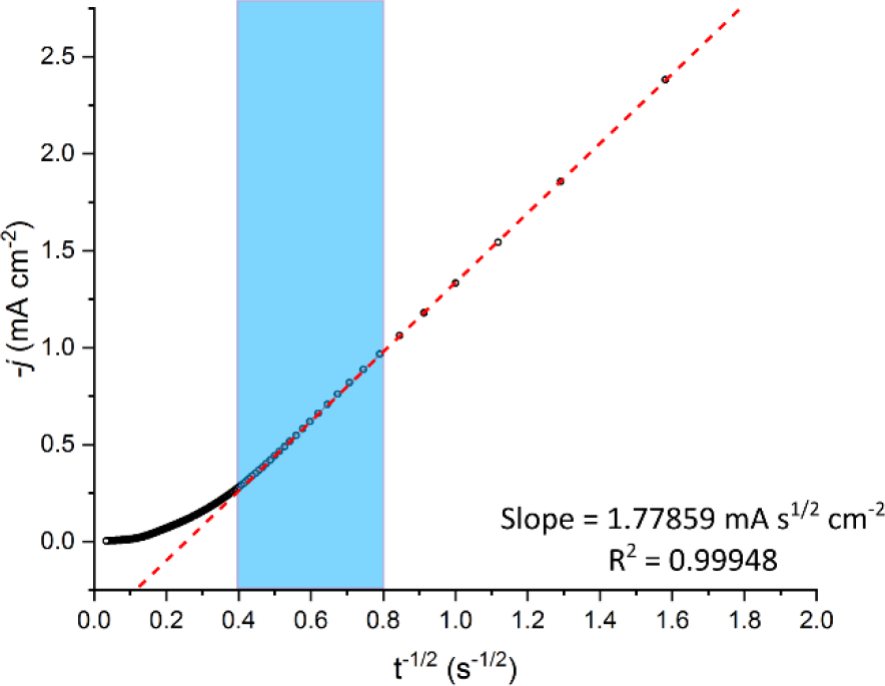
Representative Cottrell plot for Zr(dcphOH-NDI)@FTO measured in 0.5 M KPF_6_ in DMF with a time step of 0.2 s. The linear fit (red line) from ∼1.6 to 6.4 s after the potential step used to extract *D*_*e*_^app^ from eq 4 (the blue box indicates the set of points used for the linear fit).

**Figure 6:**
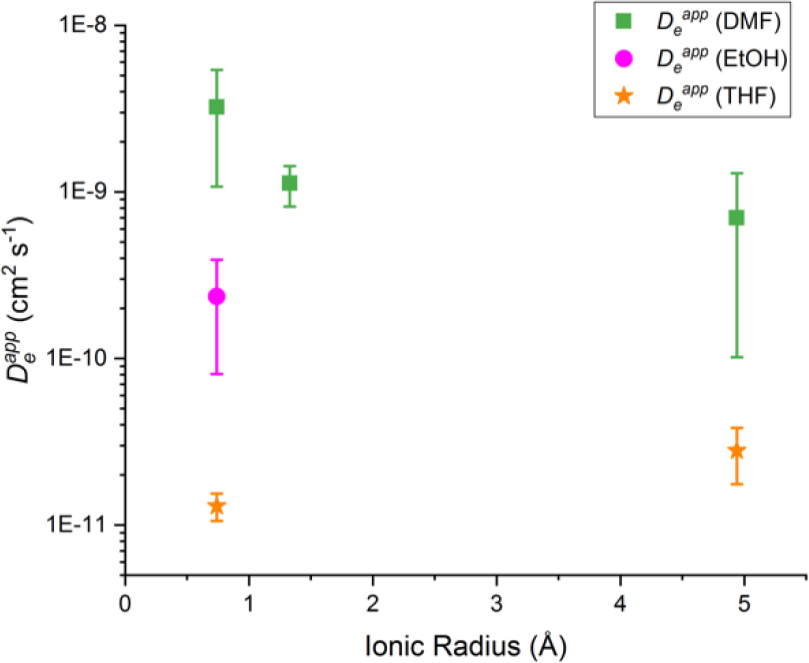
Average *D*_*e*_^app^ plotted *vs* the ionic radius of the cations employed in this study.

A representative Cottrell plot from a chronoamperometry experiment for a Zr(dcphOH-NDI)@FTO film measured in 0.5 M KPF_6_ in DMF is shown in Figure 5 to illustrate an example of the acquired data and time transients considered for *D*_*e*_^app^ determination. The average calculated apparent diffusion coefficients for Li^+^-, K^+^-, and TBA^+^-electrolytes in DMF, EtOH, and THF (when soluble) are plotted in Figure 6 and detailed in Table 3 along with the electroactive NDI surface concentrations for each of the samples.

Focusing first on the *D*_*e*_^app^ values that were obtained for different electrolytes in DMF (green points in Figure 6), the determined *D*_*e*_^app^ increases with the cation order TBA^+^ < K^+^ < Li^+^. This trend could suggest the hypothesis that in a solvent with a high dielectric constant (like DMF), the ion pairing is relatively weak, and transport of the counter ion through the MOF film is mostly influenced by the size of the diffusing cation.

When the solvent medium is changed from DMF to EtOH, the *D*_*e*_^app^ for Li^+^ decreases by approximately 1 order of magnitude (pink point in Figure 6). This decrease is consistent with stronger ion pairing of the Li^+^ cation to the reduced NDI^·–^ caused by the lower dielectric constant of the solvent. The ion pairing results ultimately in slower charge transport through the MOF film under reducing potentials.

Finally, the diffusion coefficients using Li^+^ and TBA^+^ electrolytes in THF are the slowest of the electrolyte/solvent combinations tested (orange points in Figure 6). THF, being the least polar of all solvents in this study, likely further increases the association of the high charge density Li^+^ with the reduced NDI^·–^ linker as compared to the situation in EtOH. The ion pairing in THF decreases the *D*_*e*_^app^ to a value that is even lower than that with TBA^+^, the diffusion-migration of which is greatly restricted by its size. The results are in line with the low current densities that are observed in the CVs of the Zr(dcphOH-NDI)@FTO thin films in THF, further supporting the notion that reduced ion flux of the charge-balancing cations within the film limit rapid charge transport.

### Computational Studies

A series of simulation models were built to shine light on the interaction between the different cations and Zr(dcphOH-NDI) in its reduced state. Molecular dynamics (MD) simulations based on a recent dummy atom description of the Zr^4+^ ions^55^ were conducted. The details of the model setup and computational methodology can be found in the Supporting Information. Every structure uses the interpenetrated framework (see the Supporting Information, Figure S10) in a repeating box (initial size of 100 Å × 90 Å × 110 Å) filled with either DMF or THF solvent molecules. To simulate the NDI radical anion state in different solvents with various kinds of ions, all linkers in the model were reduced to the NDI^·–^ state, and the three counter ions (Li^+^, K^+^, and TBA^+^) were added to the simulations to neutralize the system. Subsequently, a series of MD simulations were performed to heat the system to 300 K and equilibrate the density of the system and the final productive sampling. These initial simulations qualitatively show the mode of interaction between the ions and the reduced MOF and they reveal differences in the mobility of the different ions that should correlate with the experimentally measured apparent diffusion coefficients.

Taking the number of cations within a radius of 6.0 Å from the NDI oxygen atoms as a criterion, it was found that all cations (Li^+^, K^+^, and TBA^+^) associate with the reduced linkers within 10 ns (the Supporting Information, Figure S13). The binding mode of TBA^+^ is qualitatively different relative to that of the two alkali ions. In DMF, there are formally more TBA^+^ than Li^+^ and K^+^ ions within 6.0 Å of the reduced NDI linker. This is, however, an effect of the size of the TBA^+^ cation, which leads to a situation in which parts of the ion are within 6.0 Å of the NDI oxygen atoms. TBA^+^ is situated in proximity to the NDI linkers but does not interact with any preferential atom. In contrast, both Li^+^ and K^+^ interact much more specifically with the oxygen atoms of the reduced NDI linkers, forming clear Li^+^/K^+^–O pairs and sometimes bridging structures where one Li^+^ or K^+^ interacts with two oxygen atoms of neighboring NDI linkers.

**Figure 7:**
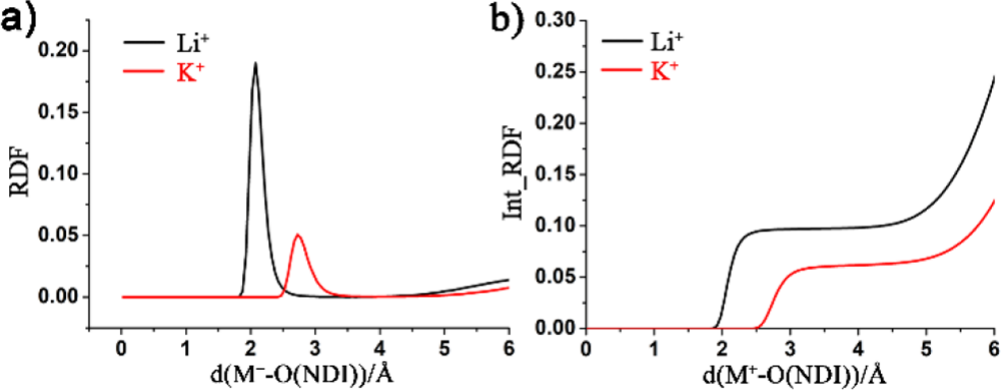
(a) Radial pair distribution functions (RDFs) and (b) integral of RDFs for the interaction between Li^+^ and K^+^ and Zr(dcphOH-NDI) in DMF. Every linker in Zr(dcphOH-NDI) is reduced by one electron. The integral RDF shows the number of ions at a distance from the NDI-*O*; as every NDI contains four *O*-centers, one cation/NDI corresponds to an integral of 0.25.

Analysis of the radial pair distribution function (RDF) for Li^+^ and K^+^ with the NDI oxygen atoms in DMF (Figure 7) shows that Li^+^ associates with a shorter distance, as expected, but also to a slightly higher extent than K^+^ as is shown by the higher value of the integral of the RDF (Figure 7b). Again, this indicates that K^+^ is less associated and thus more mobile than Li^+^ in DMF, which is at odds with the higher experimental *D*_*e*_^app^ for Li^+^. These simple calculations thus suggest that differences in ion flux that depend on the strength of ion pairing interactions cannot be the only factors that determine the speed of charge propagation through the MOF.

Simulations in less polar THF show that all three cations bind tightly with the reduced linkers and that the root-mean-square deviation (RMSD), which is a measure of cation displacement as a function time, is significantly lower for the cations in THF compared to those in DMF (see the Supporting Information, Figure S16). In THF, the integral of the cation to NDI-*O* RDFs is significantly higher, further showing the strong association of the ions to the linkers. This strong binding is consistent with the smaller experimentally determined *D*_*e*_^app^ for Li^+^ in THF, while that of TBA^+^ is further determined by the large size of the TBA^+^ cation.

**Figure 8:**
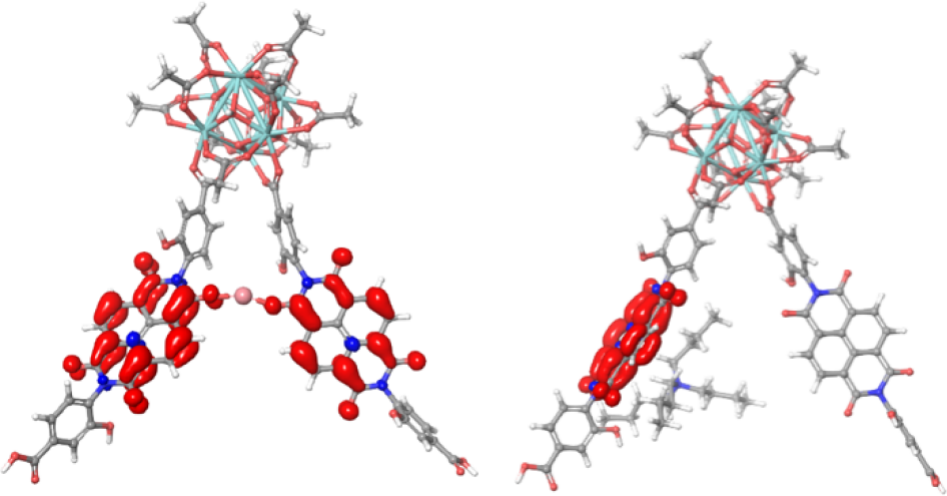
Left: a singly reduced Zr_6_O_4_(OH)_4_(OAc)_10_(NDI-OH)_2_ model system with a bridging Li^+^ counter ion. Right: a singly reduced Zr_6_O_4_(OH)_4_(OAc)_10_(NDI-OH)_2_ model system with a TBA^+^ counter ion. Spin densities (in red) illustrate electron (de)localization in the model systems.

The discrepancy between Li^+^ being engaged in the strongest ion pairing with the reduced linker, while still showing the highest *D*_*e*_^app^, warranted further investigations. While slow cation transport as a result of the ion pairing may result in higher *D*_*e*_^app^ as discussed in the introduction around Figure 1b, we hypothesized that also specific interactions between a reduced and a neutral linker could be relevant to the macroscopic *D*_*e*_^app^. For this purpose, DFT calculations at the B3LYP-D3/LACVP** level were performed on the basis of the Zr_6_O_4_(OH)_4_ cluster that were cut out from the last snapshot of the MD simulations. The detail of the DFT calculation setup is presented in the Supporting Information. The terminal carboxylate groups of the NDI linker were protonated to neutralize the charge, and the positions of the carbon atoms in the two terminal NDI-carboxylates were locked in the optimized geometry. One K^+^ or Li^+^ was placed in between two NDI oxygen atoms to form a bridge; the charge was neutral and the spin multiplicity is two, which corresponds to one reduced and one neutral linker. A structure with TBA^+^ was also optimized. Since TBA^+^ does not coordinate to the oxygen, the ion was placed close to the reduced NDI linker. Then, the structures were optimized using DFT. While both alkali ions bridge the two linkers, the interaction with Li^+^ is more specific. The Li^+^ ion forms a close-to-linear bridge (∠O–Li–O = 161°) with equal distances of 1.8 Å to each of the two oxygen atoms, leading to an O–O distance of 3.5 Å. This geometry will have two favorable effects on the electron transfer rate: (1) the distance between the donor and acceptor is as short as it can be in the framework, and (2) the geometric rearrangement involved in the electron transfer is minimal since the ion does not need to move significantly. The K^+^ ion, on the other hand, does not form the linear coordination structure, and also interacts with the phenyl ring of the linker. The K^+^–O distances are 2.6 and 2.7 Å and the O–O distance is slightly longer than for Li^+^ (4.2 Å). We found that the natural atomic charges of K and Li was 0.95 and 0.90, respectively, indicating mainly electrostatic interaction between the linkers and ions. The TBA^+^ interaction is qualitatively different in that it is positioned in proximity to one of the NDIs and does not form a bridge. A plot of the spin density of the two distinct systems with either Li^+^ or TBA^+^ as the counter ion allows a striking observation. In the bridging Li^+^ case, the spin is delocalized over the two NDI linkers, while in the TBA^+^ case, it is localized on the fragment close to the cation (Figure 8). For complete transfer, the cation will need to detach from one NDI to localize the charge. The structure with a Li^+^ ion bridging between the two NDIs in the singly reduced system is highly suggestive of a cation-coupled electron transfer reaction that proceeds in a concerted fashion, according to the diagram in Figure 1d. For the TBA^+^ system, the electron is fully localized on one fragment, and the electron transfer is more likely to occur by sequential paths. Another way of interpreting the delocalized *vs* localized electron is that the systems with localized electrons follow an outer-sphere electron hopping path, while the system with the bridging ion could proceed via a mechanism that more resembles an inner-sphere mechanism. In analogy with inner-sphere mechanisms at metal complexes, the electron transfer rate is determined by the atomic positions and by transition states where the nuclear positions determine the location of the electron.

## Conclusions

The present study is the first of its kind that systematically investigates the effect of counter ion size, ion pairing, and solvent polarity on electron hopping charge transport in MOFs. Macroscopic experimental findings are evaluated against a number of conceivable microscopic models. Experimental and computational results indicate that reduced linkers engage in ion pairing with high charge density cations, the degree of which is dependent on solvent polarity and cation size. Of the cations studied, Li^+^ engages in the strongest ion pairing, even in polar DMF solvent. Nevertheless, the Li^+^/DMF system exhibits the fastest *D*_*e*_^app^, indicating that charge propagation under these conditions is not limited by the ion-pairing association equilibrium constant *K*_eq_ = *k*_A_/*k*_D_ as outlined in Figure 1c. Instead, computational DFT results suggest that charge propagation proceeds through an unusual configuration in which a Li^+^ cation resides symmetrically between a reduced and a ground state NDI linker. In fact, the spin in this Li^+^-bridged (dcphOH-NDI)_2_ dimer is fully delocalized between the two linkers, making this configuration set up for a concerted cation-coupled electron transfer, as suggested in Figure 1d. This finding illustrates that charge propagation in a MOF that is seemingly set up for electron hopping charge transport may actually promote charge transport by a through-bond mechanism under certain conditions.

Indications of electron transport through MOFs based on a microscopic model that includes concerted cation-coupled electron transfer reactions is, to the best of our knowledge, unprecedented. The model, however, bears strong similarities to proton-coupled electron transfer processes that are a subject of intense interest.^56,57^ With the rate of charge diffusion through MOFs being highly relevant to future electronics and electrocatalysis applications, the work presented herein breaks new ground for designing materials with superior transport properties in the future. A thorough understanding of the microscopic mechanisms that are behind macroscopic transport phenomena is at the heart of this quest.

## Supporting Information Available

Detailed experimental procedures, crystal structure determination, and computational details (PDF)

## Accession Codes

CCDC 2119570 contains the supplementary crystallographic data for this paper. These data can be obtained free of charge via www.ccdc.cam.ac.uk/data_request/cif, or by emailing data_request@ccdc.cam.ac.uk, or by contacting The Cambridge Crystallographic Data Centre, 12 Union Road, Cambridge CB2 1EZ, UK; fax: +44 1223 336033.

## Abbreviations

3DED: three-dimensional electron diffraction
CV: cyclic voltammetry
DFT: density functional theory
DMF: *N,N*-dimethylformamide
DPV: differential pulse voltammetry
EtOH: ethanol
FTO: fluorine-doped tin oxide
MD: molecular dynamics
MOF: metal–organic framework
NDI: naphthalene diimide
PIZOF: porous interpenetrated Zr–organic framework
RDF: radial pair distribution function
RMSD: root-mean-square deviation
SBU: secondary binding unit
TBA^+^: *n*-tetrabutylammonium
THF: tetrahydrofuran
