# Supplementary material for: Microscopic Insights into Cation-Coupled Electron Hopping Transport in a Metal–Organic Framework
Source: J Am Chem Soc. 2022 Mar 24;144(13):5910–20. doi: 10.1021/jacs.1c13377 (PMC8990995; doi:10.1021/jacs.1c13377)
Supplement: Supplementary file 1 — ja1c13377_si_001.pdf [file ja1c13377_si_001.pdf]

Supporting Information

Microscopic insights into cation-coupled electron hopping transport in a metal-organic framework

Ashleigh T. Castner,[a] Hao Su,[b] Erik Svensson Grape,[c] A. Ken Inge,[c] Ben A. Johnson,[a]* Mårten S. G. Ahlquist,[b]* Sascha Ott[a]*

[a]Department of Chemistry - Ångström Laboratory, Uppsala University, Box 523, 75120 Uppsala, Sweden.
[b]Department of Theoretical Chemistry and Biology, KTH Royal Institute of Technology, 10691 Stockholm, Sweden.
[c]Department of Materials and Environmental Chemistry, Stockholm University, 106 91 Stockholm, Sweden.

Table of Contents

1) General Materials and Experimental Methods 2
- dcphOH-NDI Linker Synthesis 2
- Zr(dcphOH-NDI)@FTO Thin Film Preparation 2
- CV and Chronoamperometry 3
- Cottrell analysis for D_e^{app} determination 3

2) Zr(dcphOH-NDI)@FTO Thin Film Characterization 5
3) Chronoamperometry and Cottrell Analysis: Zr(dcphOH-NDI)@FTO 6
- Zr(dcphOH-NDI)@FTO: $LiClO_4$ / DMF 6
- Zr(dcphOH-NDI)@FTO: $LiClO_4$ / EtOH 7
- Zr(dcphOH-NDI)@FTO: $LiClO_4$ / THF 8
- Zr(dcphOH-NDI)@FTO: KPF_6 / DMF 9
- Zr(dcphOH-NDI)@FTO: $TBAPF_6$ / DMF 10
- Zr(dcphOH-NDI)@FTO: $TBAPF_6$ / THF 11

4) Three-dimensional electron diffraction measurements 12
- Comparison PXRD: Experimental v. Simulated from 3DED 13

5) Molecular Dynamics and Simulation Methods 13
- System Setup 13
- MD Simulations 15
- Simulation Results and Discussions 15
- DFT setup for single cluster with two linkers 18

6) References 19
7) Mol2 file: Reduced NDI 21

1) General Materials and Experimental Methods

All solvents and chemicals were purchased from commercial suppliers (Sigma Aldrich, VWR, Fluorochem, TCI) and were used without further purification unless otherwise noted. $TBAPF_6$ was recrystallized from EtOH and dried under vacuum prior to electrochemical experiments.

^{1}H NMR spectra were measured on a JEOL 400 MHz spectrometer at 293K. The chemical shifts are reported in ppm and are internally referenced to the residual solvent peak. Scanning electron microscopy (SEM) images were obtained on a Zeiss LEO 1550 Schottky FEG scanning electron microscope equipped with InLens detector at an acceleration voltage of 3 kV. Surface area and film thickness of each individual film was determined using ImageJ[1].

Cyclic voltammetry (CV) and chronoamperometry was performed using an Autolab PGSTAT100 potentiostat with GPES 4.9 software (EcoChemie). A 0.5 M solution of electrolyte in DMF, EtOH, or THF was used as the supporting electrolyte. A one-compartment, 3-electrode setup was employed with a glassy carbon (GC) rod counter electrode and a nonaqueous Ag/Ag(NO_3) (10 mM in MeCN) reference electrode, referenced to the ferrocenium/ferrocene couple ($Fc^{+/0}$). The counter and reference electrodes were isolated from the main solution by salt bridges with glass frit tips and filled with the supporting electrolyte solution. FTO with solvothermally-grown MOF thin films were employed as the working electrode. Solution-phase measurements of the linker were performed at a 1 mM concentration (unless otherwise noted) in DMF, EtOH, or THF with 0.5 M supporting electrolyte using a GC working electrode (0.071 cm^2). Prior to measurements, the solutions were sparged with Ar to remove O_2. CVs of MOF-modified FTO were measured in 7 mL of supporting electrolyte solution at a scan rate of 50 mV s^{-1}. CVs of homogeneous linker were measured in 5 mL of supporting electrolyte solution at a scan rate of 50 mV s^{-1}.

<u>Synthesis of dcphOH-NDI[2]:</u> 1,4,5,8-Naphthalenetetracarboxylic dianhydride (0.804 g, 3.0 mmol) and 4-amino-3-hydroxy benzoic acid (1.01 g, 6.6 mmol) were added to a 50 mL flame-dried, Ar-purged 2-neck RBF fitted with a reflux condenser. The solids were suspended in anhydrous DMF (20 mL). The reaction was stirred in a 145°C sand bath overnight under Ar. The reaction was then allowed to cool to RT, and 5 mL of 1M HCl was added to the solution. The acidified mixture was slowly added to ice cold H_2O (250 mL) to precipitate the product as a pale yellow-brown solid. The solids were collected by centrifugation, and the supernatant decanted. The solids were washed sequentially with EtOH (50 mL), H_2O (50 mL), and Et_2O (50 mL). The resulting solid was dried under vacuum to yield the product as a yellow-brown solid. Yield: 1.367 g (84%) ^{1}H NMR (400 MHz, DMSO-d_6): 10.17 (m, 2H); 8.75 (s, 4H); 7.57-7.60 (m, 2H); 7.52-7.57 (m, 2H); 7.45-7.50 (m, 2H).

<u>Zr(dcphOH-NDI)@FTO thin film preparation:</u> MOF thin films on FTO were prepared similarly as previously described[2]. FTO (cut into ~1x2 cm pieces) was washed by sequential sonication in 1% Alconox in deionized H_2O, absolute EtOH, and acetone (20 minutes each wash) and dried under a stream of N_2. The cleaned FTO was immersed in a 1 mM solution of the dcphOH-NDI linker in DMF (anhydrous) overnight to form a self-assembled monolayer (SAM). The SAM-modified FTO was then rinsed with DMF and soaked in DMF while preparing the MOF solutions. To prepare the MOF-synthesis solution, $ZrCl_4$ (23.3 mg, 0.10 mmol), dcphOH-NDI (53.8 mg, 0.10 mmol), glacial acetic acid (286 µL, 5 mmol), and DMF (8 mL), were added to a 20 mL vial and sonicated to dissolve the solids. The SAM-modified FTO was then dried under a stream of N_2, and ½ of the FTO carefully wrapped with Teflon tape, leaving ~1 cm^2 of FTO exposed. The SAM-modified FTO was then immersed in the MOF synthesis solution and propped against the vial wall so that the FTO was tilted in the vial (Teflon-side at the vial bottom, FTO side facing up toward the bulk solution). The vial was then incubated in a sand bath in a 120°C oven for 72 hours to allow the MOF film to grow solvothermally. After this incubation, the MOF-modified FTO substrates were removed from the synthesis solution,

and the Teflon tape carefully unwrapped. The modified FTO was then rinsed with fresh DMF and soaked in DMF until needed for analysis.

<u>CV and Chronoamperometry of Zr(dcphOH-NDI)@FTO:</u> MOF films not analyzed in DMF were soaked in the solvent of the electrochemical analysis for at least 1 hour to exchange the DMF from the film. After taking a background CV of the electrolyte solution with a glassy carbon disk (0.071 cm^2) working electrode to verify the system was purged of O_2, a Zr(dcphOH-NDI)@FTO sample was removed from the soaking solvent and dried under a N_2 stream. The MOF-modified electrode was then immediately mounted into a sample holder and wrapped with Teflon tape, leaving ~1 cm^2 of MOF thin film exposed. The electrode was then immediately introduced into the degassed electrolyte solution and left to soak for ~10 minutes while continuing to stir and degas the electrolyte. After this soaking period, the MOF film was cycled for 50 CV scans at 50 mV s^{-1} to equilibrate the film, by which point the current response was stabilized for the final successive scans. For samples analyzed in DMF, CVs showed two distinct redox features corresponding to the $NDI^{0/\bullet-}$ and $NDI^{\bullet-/2-}$ couples. From these CVs, a potential lying between the redox couples was chosen to isolate the $NDI^{0/\bullet-}$ redox event for chronoamperometry. For samples analyzed in THF and EtOH, the measured CVs show either one broad redox feature (Li^+/THF, Li^+/EtOH) or a poorly defined feature (TBA^+/THF) in the expected potential window for both the $NDI^{0/\bullet-}$ and $NDI^{\bullet-/2-}$ couples. To resolve the two redox events and choose an appropriate potential to isolate the $NDI^{0/\bullet-}$ couple for controlled potential experiments, differential pulse voltammetry (DPV) from 0 V → -1.7 V (unreferenced) was applied with an interval time of 0.1 s, and step potential of 1 mV. Controlled potential experiments were performed by first applying a non-reducing potential (determined from MOF film CVs, generally ~ -0.6 V v. $Fc^{+/0}$) for 90s to ensure the NDI linkers in MOF film are in the NDI^0 state. The potential was then stepped down to an appropriate potential to generate the NDI radical anion ($NDI^{\bullet-}$) and this potential then held for 900s. The potential was then stepped back to a non-reducing potential for 90s to return the film to a neutral state. After chronoamperometry, 3 consecutive CV scans (scan rate: 50 mV s^{-1}) were taken of MOF-modified electrodes to verify stability of film throughout controlled potential experiments.

<u>Cottrell analysis for D_e^{app} determination to assess cation diffusion-migration in MOF films:</u>

The electroactive concentration of NDI linkers in each MOF thin film and the apparent diffusion coefficient (D_e^{app}) in the different electrolyte/solvent combinations were determined by chronoamperometry. The data for all analyzed Zr(dcphOH-NDI)@FTO films are shown in Figures S2-S7.

After applying a sufficiently mild potential for 90s to begin with a neutral film, the potential was stepped to an appropriately negative potential to reduce the NDI linkers to the $NDI^{0/\bullet-}$ state and held for 900s. The current density (*j*) and recorded charge (Q) from this potential step were plotted vs. time to determine the total charge passed. A residual background current can be observed as the recorded charge does not decay to a slope of 0 after an extended time. This background current is subtracted by fitting the linear region of the charge (t = 400-600s after potential step for fits shown). The y-intercept of this linear fit line corresponds to the total charge passed to reduce all electrochemically addressable NDI linkers to the $NDI^{\bullet-}$ redox state in the film. The electroactive NDI concentration[3] was calculated according to:

$$\Gamma_e = \frac{|Q|}{nFS_A}$$

Where Γ_e is the electroactive linker concentration (in mol cm^{-2}), Q is the charge (in C), n is the number of electrons involved in the redox event, F is Faraday's constant (in C mol^{-1}), and S_A is the surface area of the MOF-modified electrode (in cm^2). Here, it is assumed that n=1 as the applied potential is chosen to isolate the $NDI^{0/\bullet-}$ couple. S_A is independently determined for each sample for accuracy.

To determine D_e^{app} for electron-hopping diffusion in each system, the time-dependent current response (here identified as time-dependent current density *j*(t)) was fitted to the Cottrell equation:

$$j(t) = \frac{nF\Gamma_e\sqrt{D_e^{app}}}{d_f\sqrt{\pi t}}$$

Where d_f is the MOF film thickness (in cm^2) and D_e^{app} is the diffusion coefficient for charge diffusion in the film (in cm^2 s^{-1}). d_f for each film is measured from SEM images taken after electrochemical measurements. From a plot of *j*(t) vs. $t^{-1/2}$, it is evident that for points corresponding to times shortly after the potential step, a linear trend is observed. These short time points correspond to a regime where the diffusion layer for electron-hopping charge diffusion is small and confined within the film, i.e. where diffusion is semi-infinite. Therefore, by fitting the linear region of this plot, D_e^{app} can be extracted from the following relationship:

$$D_e^{app} = \left(\frac{Slope * d_f\sqrt{\pi}}{nF\Gamma_e}\right)^2$$

Experimentally determined values of Γ_e and D_e^{app} for each individual film measured in the noted electrolyte solutions are detailed in Tables S2 – S7 below the correspond CV/chronamperometry data and Cottrell analysis. Averaged values for each set of supporting electrolyte solution conditions (n=3) is given in the main text (Table 3).

2) Zr(dcphOH-NDI)@FTO Thin Film Characterization

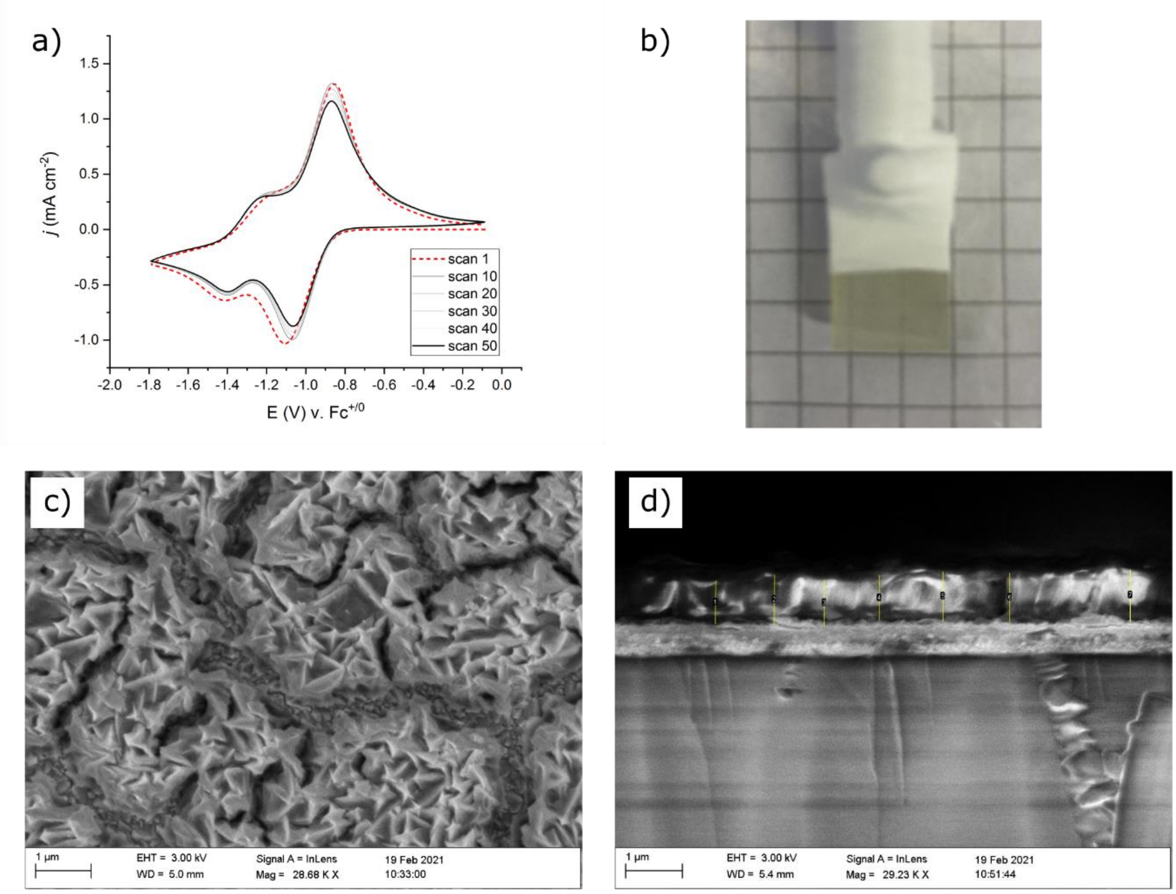

Figure S1. Representative characterizations and measurements of Zr(dcphOHNDI)@FTO. (a) Initial CV cycling of Zr(dcphOH-NDI)@FTO measured in 0.5 M KPF_6 in DMF (v = 50 mV s^{-1}), demonstrating redox behavior. CV cycling previously described for these MOF films demonstrated a 'conditioning' effect, with increasing current until a maximum current density was reached. This effect was not typically observed for all films measured in this study, likely due to the film handling prior to electrochemical analysis. For all samples reported here, films were kept solvent-swelled at all times and only dried under a stream of N_2 directly before introducing to cell. This treatment likely prevents desiccation of the MOF film that must be 'reswelled' during the initial CV cycling period, leading to improved current responses as a result of optimized pore accessibility due to the likelihood of minimal pore collapse. (b) Image of film before introducing to electrochemical cell. Segment lengths of grid paper measure 0.5 cm. Surface area (S_A) determined from this image using ImageJ.[1] (c) SEM image of Zr(dcphOH-NDI)@FTO to verify similarity to previously reported materials. SEM confirms PIZOF morphology of MOF films. Cracks in the film are likely due to desiccation of the MOF film when under vacuum in the SEM chamber, leading to collapse of individual MOF particles comprising film. Films were not sputter-coated. (d) SEM image of cross section of Zr(dcphOH-NDI)@FTO. Film thickness (d_f) determined from cross section images using ImageJ[1], with multiple measurements depicted (yellow lines) and averaged for final value of d_f.

3) Chronoamperometry and Cottrell Analysis: Zr(dcphOH-NDI)@FTO

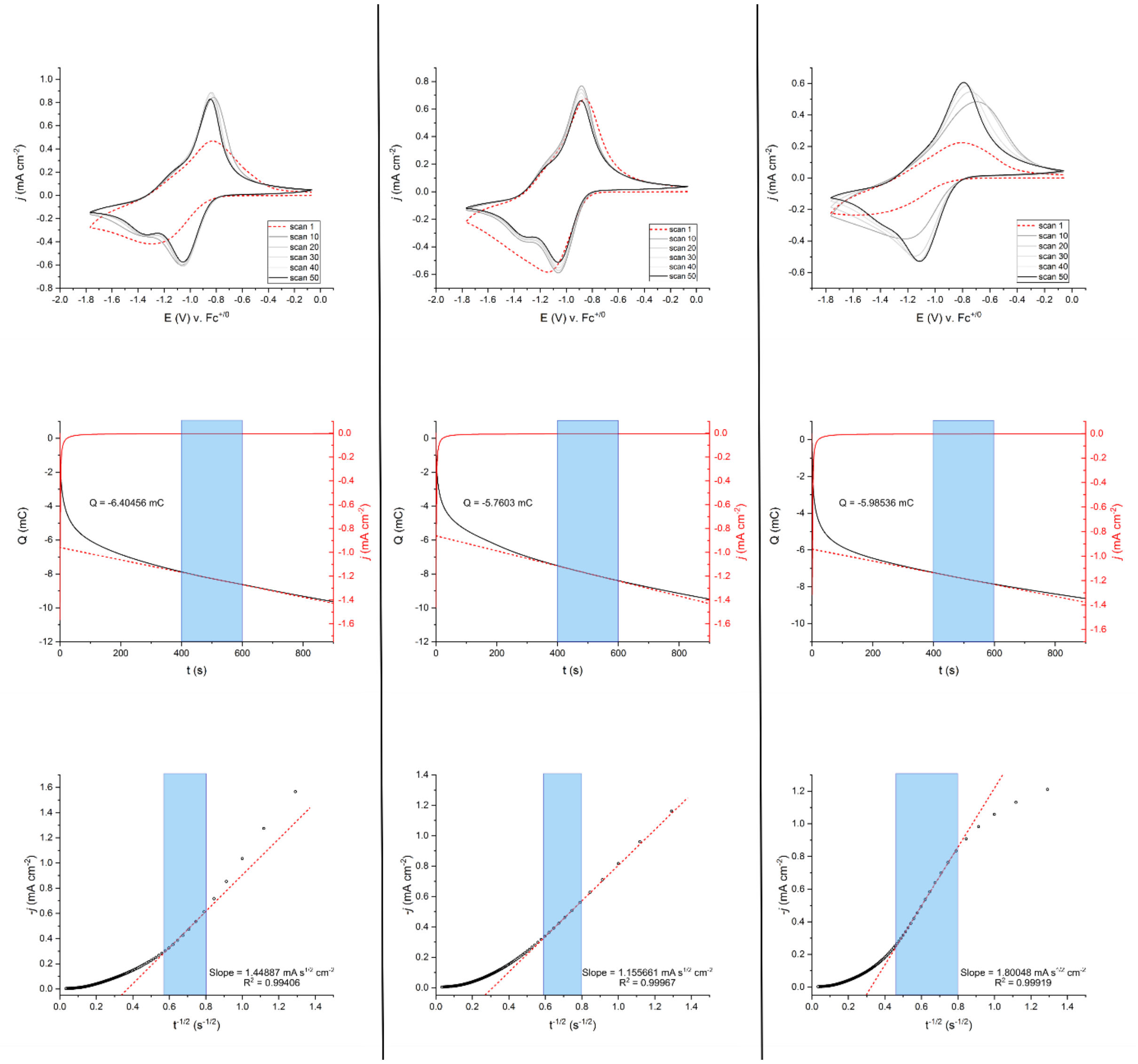

Figure S2. Electrochemical data for all Zr(dcphOH-NDI)@FTO samples measured in 0.5 M $LiClO_4$ in DMF. Top row: CV cycling (50 scans) for all samples at scan rate of 50 mV s^{-1}. Middle row: chronoamperometry (solid red line) and chronocoulometry (solid black line) after stepping the potential to isolate the $NDI^{0/\bullet -}$ redox couple for each sample (selected from corresponding CVs, generally ~ -0.67 V → -1.22 V v. $Fc^{+/0}$). The total charge passed after complete reduction for each sample was determined after subtracting a residual background current (red dashed line). Blue boxes illustrate the window of time points chosen for linear fits. Bottom row: Cottrell plots of MOF film samples after potential step. Linear fits are shown by red dashed lines, and blue boxes illustrate the window of points selected for linear fits.

Table S1. Table of parameters and experimentally measured Γ_e and D_e^{app}: $LiClO_4$ in DMF

	S_A (cm²)	d_f (µm)	Q (mC)	Cottrell Slope	Γ_e (mol cm⁻²)	D_e^{app} (cm² s⁻¹)
Film 1	1.130	1.458	-6.405	1.287	5.874e-8	3.441e-9
Film 2	1.053	0.835	-5.760	1.156	5.670e-8	9.775e-10
Film 3	0.966	1.411	-5.985	1.800	6.422e-8	5.278e-9

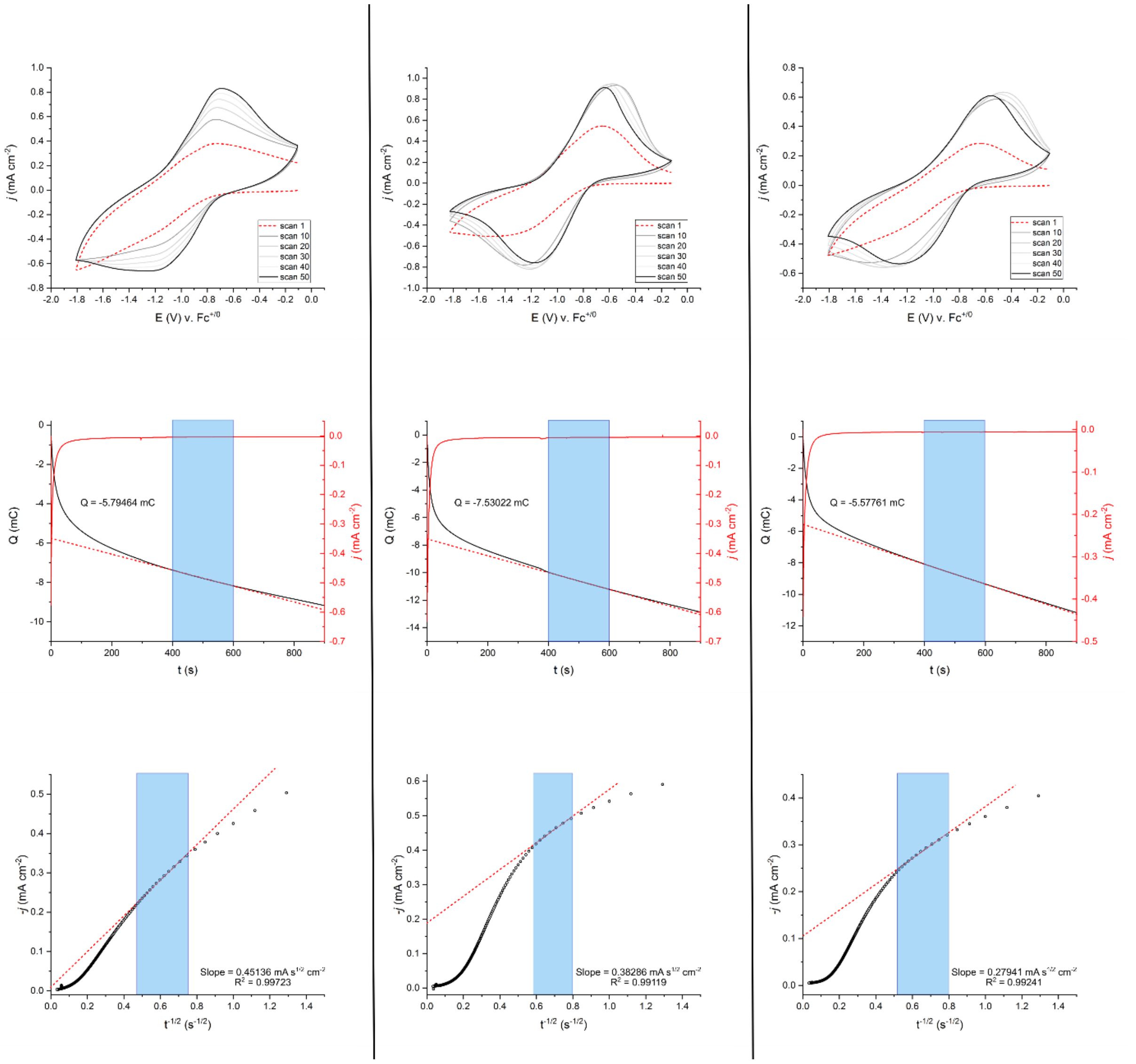

Figure S3. Electrochemical data for all Zr(dcphOH-NDI)@FTO samples measured in 0.5 M $LiClO_4$ in EtOH. Top row: CV cycling (50 scans) for all samples at scan rate of 50 mV s^{-1}. Middle row: chronoamperometry (solid red line) and chronocoulometry (solid black line) after stepping the potential to isolate the $NDI^{0/\bullet-}$ redox couple for each sample (selected from DPV, generally ~ -0.56 V → -0.94 V v. $Fc^{+/0}$). The total charge passed after complete reduction for each sample was determined after subtracting a residual background current (red dashed line). Blue boxes illustrate the window of time points chosen for linear fits. Bottom row: Cottrell plots of MOF film samples after potential step. Linear fits are shown by red dashed lines, and blue boxes illustrate the window of points selected for linear fits.

Table S2. Table of parameters and experimentally measured Γ_e and D_e^{app}: $LiClO_4$ in EtOH

	**S_A (cm^2)**	**d_f (µm)**	**Q (mC)**	**Cottrell Slope**	**Γ_e (mol cm^{-2})**	**D_e^{app} (cm^2 s^{-1})**
Film 1	0.951	0.963	5.795	0.451	6.315e-8	1.599e-10
Film 2	1.174	1.092	7.530	0.383	6.648e-8	1.335e-10
Film 3	1.181	1.941	5.578	0.279	4.895e-8	4.144e-10

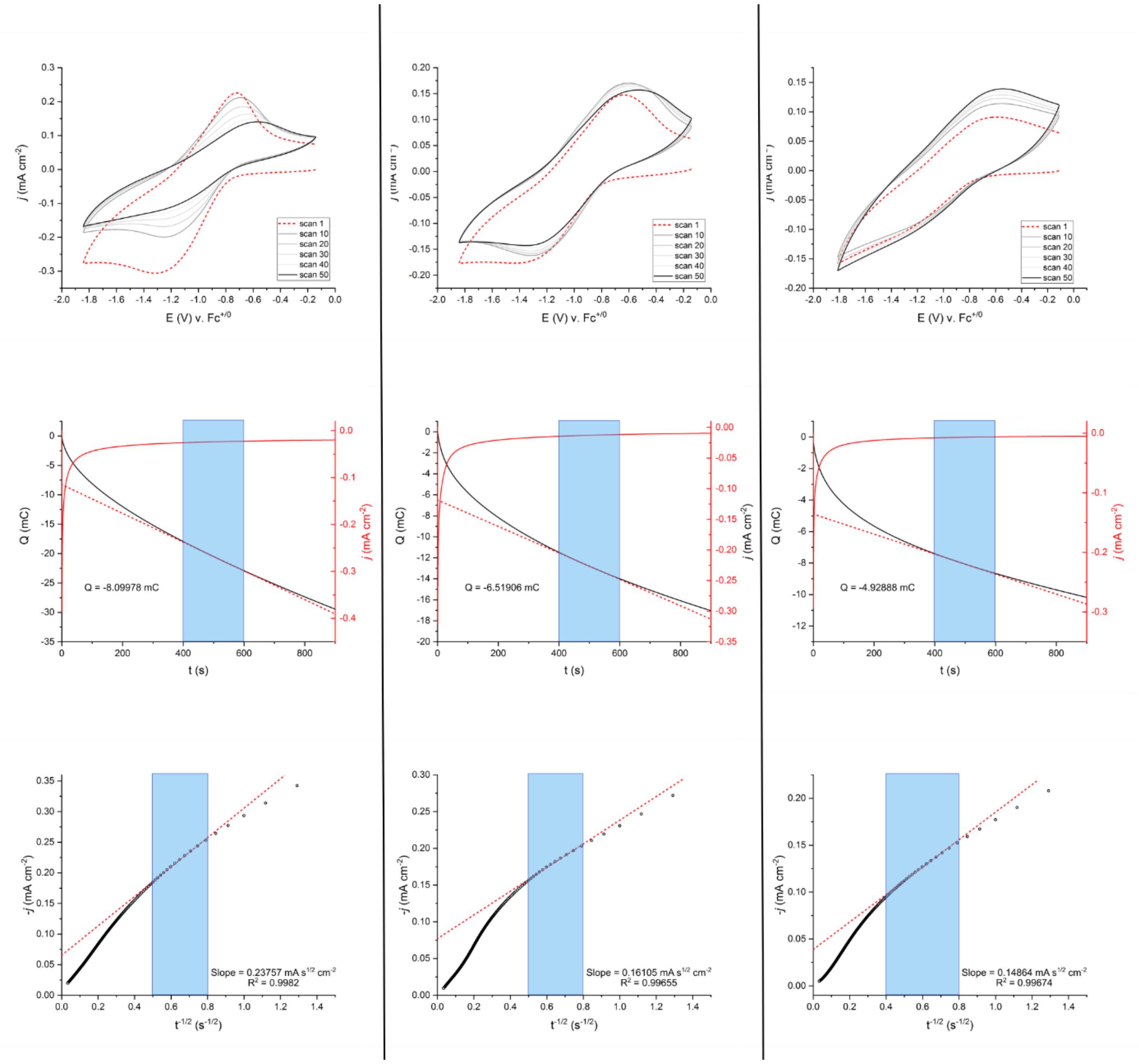

Figure S4. Electrochemical data for all Zr(dcphOH-NDI)@FTO samples measured in 0.5 M $LiClO_4$ in THF. Top row: CV cycling (50 scans) for all samples at scan rate of 50 mV s^{-1}. Middle row: chronoamperometry (solid red line) and chronocoulometry (solid black line) after stepping the potential to isolate the $NDI^{0/\bullet-}$ redox couple for each sample (selected from DPV, generally ~ -0.59 V → -1.40 V v. $Fc^{+/0}$). The total charge passed after complete reduction for each sample was determined after subtracting a residual background current (red dashed line). Blue boxes illustrate the window of time points chosen for linear fits. Bottom row: Cottrell plots of MOF film samples after potential step. Linear fits are shown by red dashed lines, and blue boxes illustrate the window of points selected for linear fits.

Table S3. Table of parameters and experimentally measured Γ_e and D_e^{app}: $LiClO_4$ in THF

	S_A (cm²)	d_f (µm)	Q (mC)	Cottrell Slope	Γ_e (mol cm⁻²)	D_e^{app} (cm² s⁻¹)
Film 1	1.044	0.705	8.100	0.238	8.041e-8	1.463e-11
Film 2	0.978	0.879	6.519	0.161	6.909e-8	1.416e-11
Film 3	0.929	0.643	4.929	0.149	5.499e-8	1.020e-11

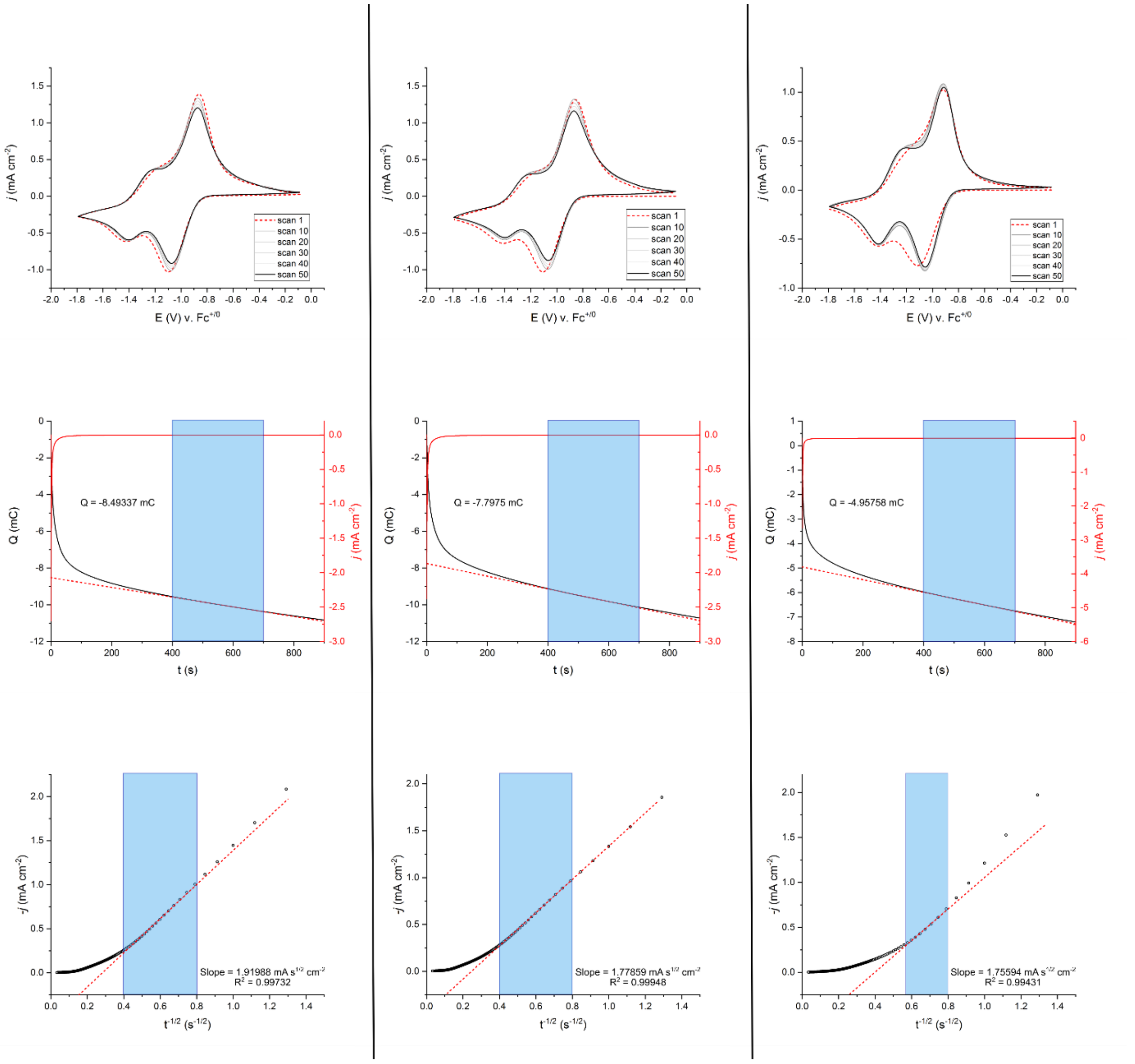

Figure S5. Electrochemical data for all Zr(dcphOH-NDI)@FTO samples measured in 0.5 M KPF_6 in DMF. Top row: CV cycling (50 scans) for all samples at scan rate of 50 mV s^{-1}. Middle row: chronoamperometry (solid red line) and chronocoulometry (solid black line) after stepping the potential to isolate the $NDI^{0/\bullet-}$ redox couple for each sample (selected from corresponding CVs, generally ~ -0.69 V → -1.27 V v. $Fc^{+/0}$). The total charge passed after complete reduction for each sample was determined after subtracting a residual background current (red dashed line). Blue boxes illustrate the window of time points chosen for linear fits. Bottom row: Cottrell plots of MOF film samples after potential step. Linear fits are shown by red dashed lines, and blue boxes illustrate the window of points selected for linear fits.

Table S4. Table of parameters and experimentally measured Γ_e and D_e^{app}: KPF_6 in DMF

	S_A (cm^2)	d_f (µm)	Q (mC)	Cottrell Slope	Γ_e (mol cm^{-2})	D_e^{app} (cm^2 s^{-1})
Film 1	1.015	0.786	8.493	1.920	8.673e-8	1.022e-9
Film 2	0.865	0.849	7.798	1.779	9.343e-8	8.815e-10
Film 3	0.893	0.683	4.958	1.756	5.754e-8	1.465e-9

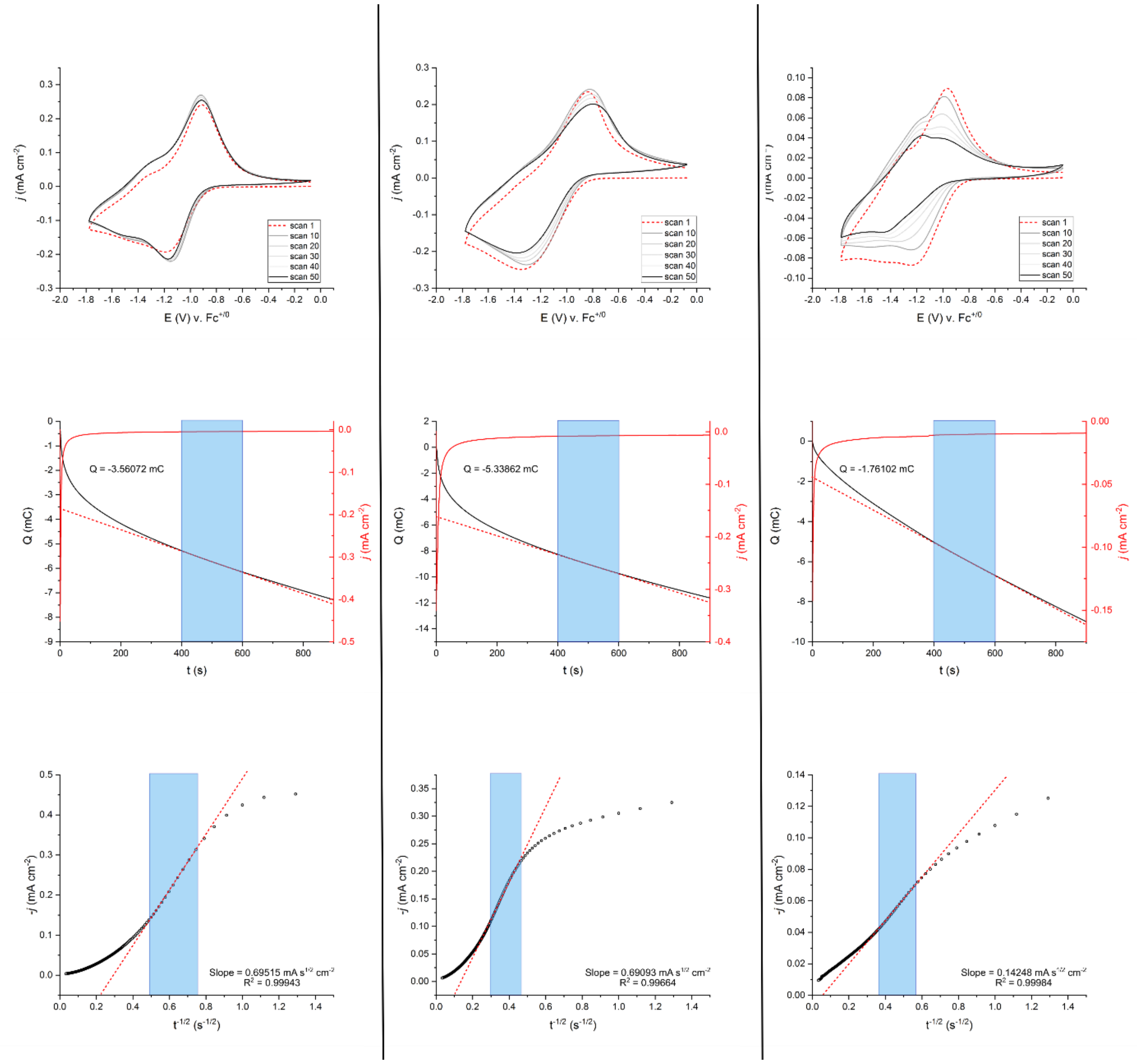

Figure S6. Electrochemical data for all Zr(dcphOH-NDI)@FTO samples measured in 0.5 M $TBAPF_6$ in DMF. Top row: CV cycling (50 scans) for all samples at scan rate of 50 mV s^{-1}. Middle row: chronoamperometry (solid red line) and chronocoulometry (solid black line) after stepping the potential to isolate the $NDI^{0/\bullet-}$ redox couple for each sample (selected from corresponding CVs, generally ~ -0.68 V → -1.38 V v. $Fc^{+/0}$). The total charge passed after complete reduction for each sample was determined after subtracting a residual background current (red dashed line). Blue boxes illustrate the window of time points chosen for linear fits. Bottom row: Cottrell plots of MOF film samples after potential step. Linear fits are shown by red dashed lines, and blue boxes illustrate the window of points selected for linear fits.

Table S5. Table of parameters and experimentally measured Γ_e and D_e^{app}: $TBAPF_6$ in DMF

	S_A (cm^2)	d_f (µm)	Q (mC)	Cottrell Slope	Γ_e (mol cm^{-2})	D_e^{app} (cm^2 s^{-1})
Film 1	1.007	1.002	3.561	0.695	3.665e-8	1.219e-9
Film 2	0.950	1.322	5.339	0.691	5.824e-8	8.303e-10
Film 3	0.791	0.605	1.761	0.142	2.307e-8	4.708e-11

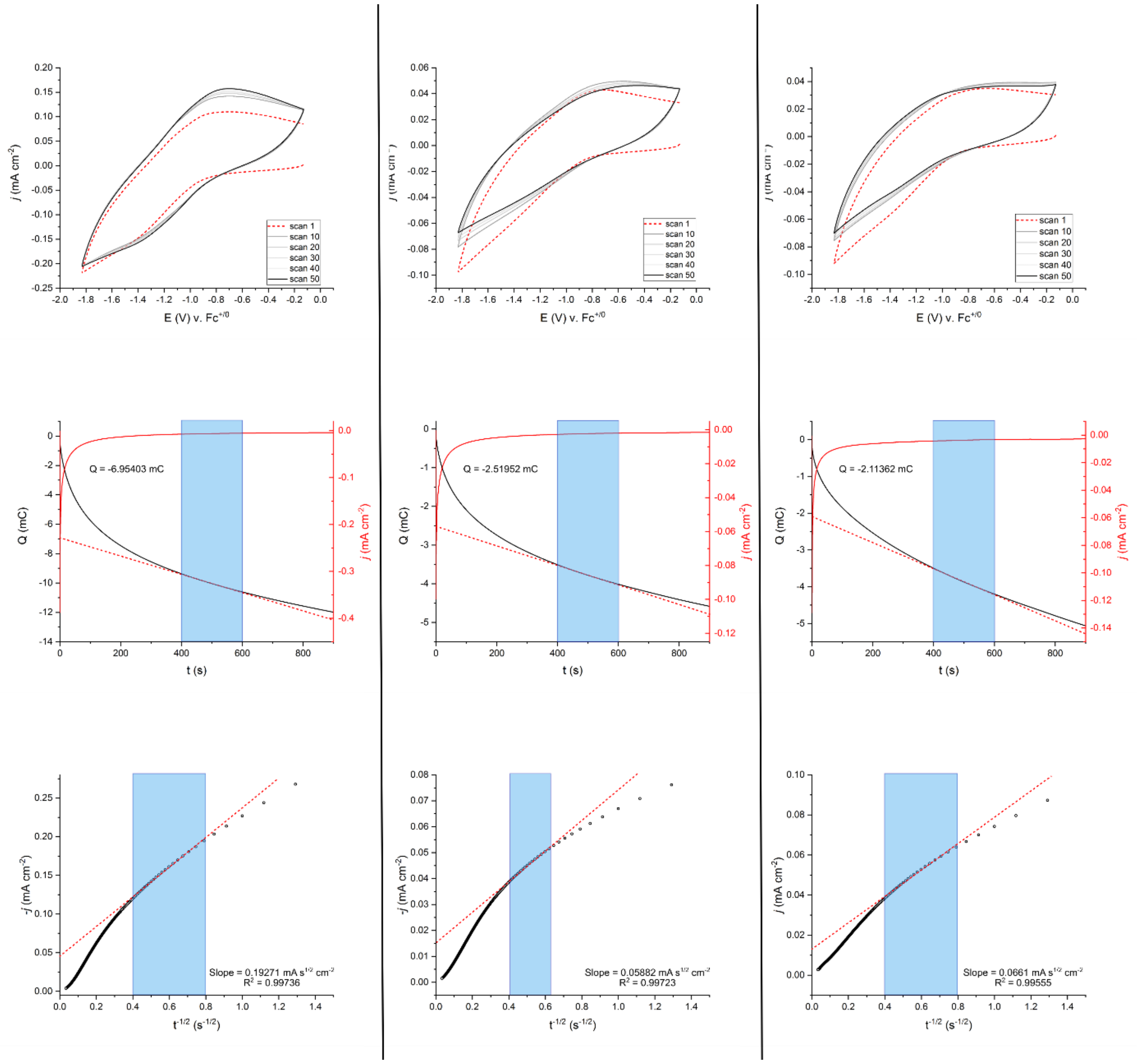

Figure S7. Electrochemical data for all Zr(dcphOH-NDI)@FTO samples measured in 0.5 M $TBAPF_6$ in THF. Top row: CV cycling (50 scans) for all samples at scan rate of 50 mV s^{-1}. Middle row: chronoamperometry (solid red line) and chronocoulometry (solid black line) after stepping the potential to isolate the $NDI^{0/\bullet-}$ redox couple for each sample (selected from DPV, generally ~ -0.73 V → -1.43 V v. $Fc^{+/0}$). The total charge passed after complete reduction for each sample was determined after subtracting a residual background current (red dashed line). Blue boxes illustrate the window of time points chosen for linear fits. Bottom row: Cottrell plots of MOF film samples after potential step. Linear fits are shown by red dashed lines, and blue boxes illustrate the window of points selected for linear fits.

Table S6. Table of parameters and experimentally measured Γ_e and D_e^{app}: $TBAPF_6$ in THF

	S_A (cm²)	d_f (µm)	Q (mC)	Cottrell Slope	Γ_e (mol cm⁻²)	D_e^{app} (cm² s⁻¹)
Film 1	1.000	1.242	6.954	0.193	7.207e-8	3.723e-11
Film 2	1.112	0.889	2.520	0.059	2.348e-8	1.674e-11
Film 3	0.947	1.042	2.114	0.066	2.313e-8	2.992e-11

4) Three-dimensional electron diffraction measurements

Samples were prepared by dispersing solvothermally-prepared bulk Zr(dcphOH-NDI) in ethanol, which was then gently mortared and drop-cast onto a copper grid covered in a holey carbon film. The sample was loaded into a Gatan 914 cryo transfer holder, which was then cooled to approximately 200 K before being inserted into the TEM column. Three-dimensional electron diffraction (3DED) data were collected using a JEOL JEM-2100 TEM at 98 K, equipped with a Timepix detector from Amsterdam Scientific Instruments, while continuously rotating the crystal at 0.45° s^{-1}. The experiment was carried out using Instamatic,[4] with data reduction performed in XDS.[5] The acquired intensities were then used to solve the structure of SU-102 with SHELXT,[6] and refined using SHELXL,[7] with electron scattering factors extracted from SIR2014.[8] From the 3DED data, all non-hydrogen atoms could be located in the initial structure solution.

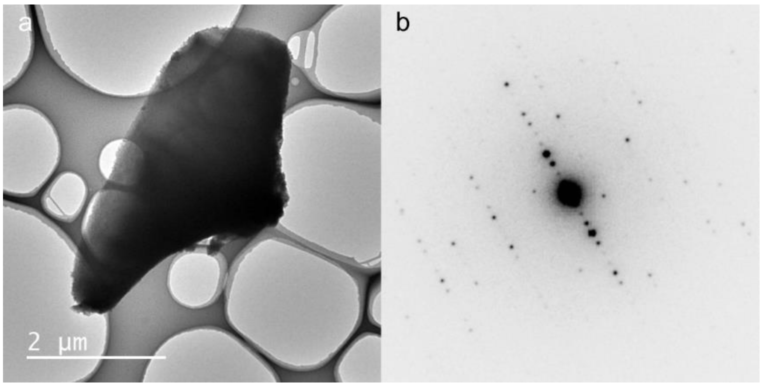

Figure S8: (a) Transmission electron microscope image of the measured Zr(dcphOH-NDI) crystal, as well as (b) a single frame from the 3DED experiment.

Table S7: Crystallographic table for electron diffraction data of Zr(dcphOH-NDI).

Empirical formula	$C_{672}N_{48}O_{272}Zr_{24}$
Wavelength	0.0251 Å
Crystal system	Triclinic
Space group	$F4_132$ (No. 210)
Unit cell dimensions	a = 39.90 Å
Volume	63516 Å^3
Z	2
Rotation range	110.43° (-54.66 to 55.77°)
Temperature	98 K
Index ranges	$-44 \leq h \leq 40$<br>$-40 \leq k \leq 48$<br>$-46 \leq l \leq 39$
Reflections collected	44132
Independent reflections	5034 [R(int) = 0.3302]
Completeness (to 0.82 Å resolution)	99.9 %
R_1 (ED model) [I > 2σ(I)]	0.2398

Due to the potential for positional disorder of the hydroxyl groups on the carboxylate-bearing phenyl rings of the dcphOH-NDI linker during diffraction measurement, the structure model of the MOF indicates a partial occupancy for each likely position of these hydroxyl groups (50% each). Because the diffraction data shows this single hydroxyl is equally likely to be located on either side of the phenyl spacer, the structures shown in **Figure 2** show only one hydroxyl group on each phenyl ring for visual clarity.

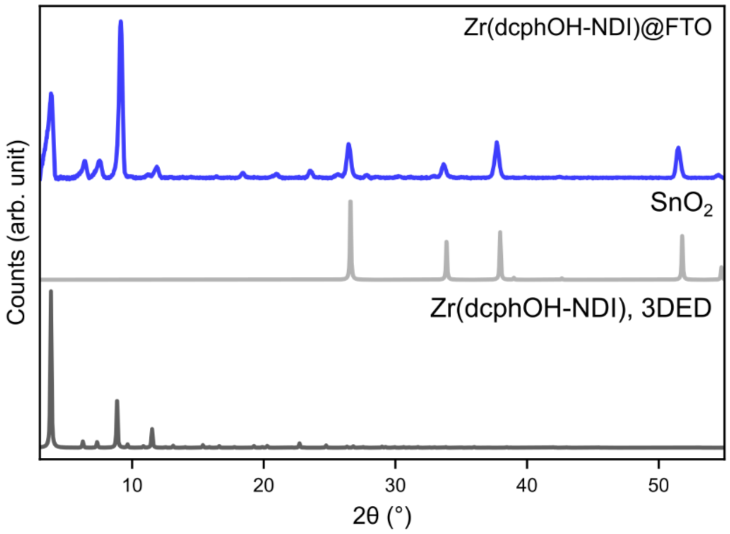

Figure S9. Comparison of PXRD patterns between the simulated powder pattern for Zr(dcphOH-NDI) as determined by 3DED (bottom, dark grey), a simulated powder pattern of tin oxide (middle, light grey) and the experimental powder pattern of Zr(dcphOH-NDI)@FTO (top, blue).

5) Molecular Dynamics and Simulation Methods

System Setup

To simulate the cation ions diffusion in different solvents, we set up a fully reduced Zr(dcphOH-NDI) system. The geometrical structure was cut from the 3 × 3 × 3 UiO-66[9] units. Then, the linker of the crystal structure of UiO-66 were substituted with the NDI-based Zr(dcphOH-NDI) linker. Subsequently, the interpenetrated framework model was built by putting part of the obtained geometrical structure into its duplicate structure. Acetate ions were used to saturate the terminate sites of the systems. The Zr^{4+} in the metal cluster of Zr(dcphOH-NDI) were described by our published dummy atom model.[10] The parameters of NDI, oxygen, hydroxyl ligands, TBA and solvent molecules dimethylformamide (DMF) and tetrahydrofuran (THF) were taken from the General Amber Force Field (GAFF2) force field.[11] The partial atomic charges of NDI and TBA were obtained by the restrained electrostatics potential (RESP)[12] method at the level of B3LYP/6-31G*[13]. The RESP charges of DMF and THF molecules were calculated at B3LYP/6-31G* level with the corresponding polarizable continuum model (CPCM)[14] as the solvent environment.

The Zr(dcphOH-NDI) model as shown in **Figure S10** was solvated in DMF or THF solvent box with the edge extend to 10 Å from the frameworks, giving a box with the size 100Åx90Å x110Å. Then, for each solvent box the systems were neutralized with K^+, Li^+ and TBA^+ cations, respectively. In order to compare the binding affinity between cation and the reduced NDI in different solvents, a series of models containing one reduced NDI and respective cations were built (illustrated in **Figure S11**. Then, the models were solvated by pre-equilibrated DMF or THF boxes giving a rectangular solved box with 20 Å from the solute molecules to the edge of the box. Solvation processes were performed by using the LEaP module in AmberTools.[15] Periodic boundary conditions (PBC) are used for all of our MD simulations.

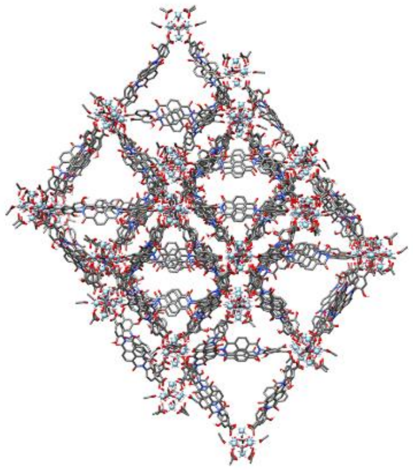

Figure S10. Graphical representation of the Zr(dcphOH-NDI) PIZOF model that was used in MD simulations for cation distribution.

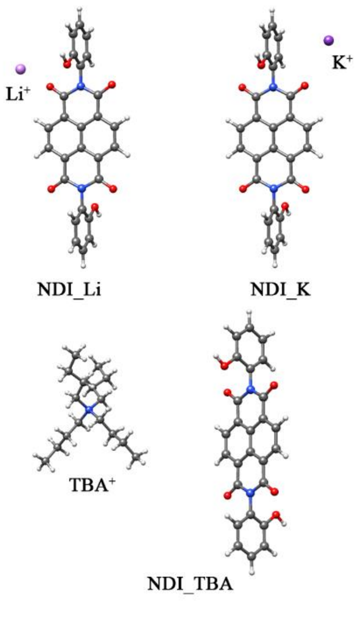

Figure S11. Individual singly reduced NDI linkers with different cations (Li^+, K^+, and TBA^+) used for the potential of mean force simulations to estimate the binding free energies of the ions to the reduced linkers in different solvents.

MD simulation

MD simulations were performed and analyzed using the AMBER16 and AmberTools packages.[15] For all of the solved systems, two stages energy minimization were implemented: first, the solvent molecules were minimized by constraining the solute molecules, and then the whole system was minimized using 5000 steps steepest conjugate and another 5000 steps for conjugate gradient. The systems were then heated from 50 to 300 K under the NVT (constant volume and temperature) ensemble in 50 ps, after which a 200 ps density equilibration MD simulation was performed under the NPT (constant pressure and temperature) ensemble for each of the systems. During the density equilibration, the Langevin thermostat[16] and Berendsen barostat[17] with a collision frequency of 2 ps and a pressure relaxation time of 1 ps were used to control the target temperature and pressure to 300 K and 1.0 atm. Thereafter, productive NPT MD simulations were performed for 30 ns and 5 ns for UiO-NDI and one-linker systems, respectively. From the last snapshot of the 5 ns MD simulations, a series of 500 ps steered MD simulations were performed to generate trajectories along the binding of cations to the reduced NDI molecule. With the obtained snapshot, umbrella sampling simulations were carried out with 0.25 Å windows separation to obtain the binding free energy profile between cations and the reduced NDI. The weighted histogram analysis (WHAM) method was used to generate the potentials of mean force.[18-20]

Simulation Results and Discussion

The measured experiment shows the cation diffusion (D_i) between different cations in same solvent only have 2-4 times different. To understand the movement difference between different cations and the effects of solvents on this movement, we performed a series of MD simulations. First, we analyzed the diffusion of cations to the reduced linkers of Zr(dcphOH-NDI).

We find that the ions bind somewhat differently to the linkers, where Li^+ and K^+ preferentially interact with the oxygen atoms of the NDI while TBA^+ interacts in a less specific manner (**Figure S12**). To illustrate the movement of cations in different solvents, we measured the variation of the number of ions within a distance (3 Å for Li^+, 3.5 Å for K^+ and 4 Å for TBA^+) of an imide-oxygen center of the NDI linker (**Figure S13**) with the simulation time. In both DMF and THF solvents, Li^+ and K^+ quickly bind (less than 2 ns) with the reduced NDI, whereas the TBA^+ needs a longer time (~10 ns) to fully associate with all the NDI linkers. This is likely due to the larger volume and the shielded charges on TBA^+. In DMF, the number of binding Li^+ is slightly higher than that of K^+, but after 5 ns they both have lower binding numbers than TBA^+. This effect is caused by TBA^+ having long side chains that bring the cation within the 6 Å distance of the NDI-O.

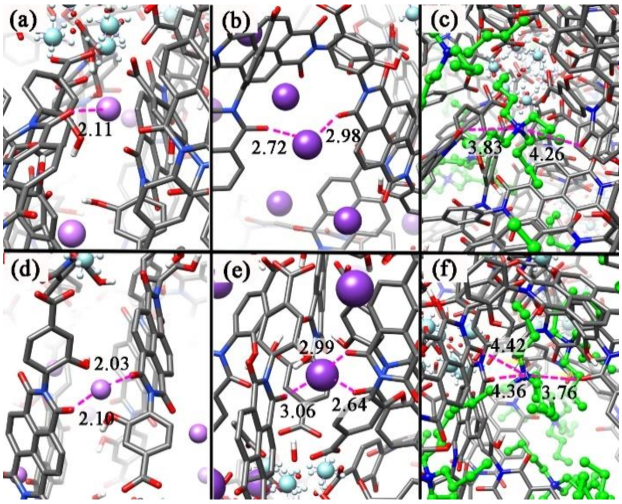

Figure S12. Snapshots from the from MD simulations illustrating the cation binding sites in the interconnected UiO-MOF: (a), (b) and (c) represent Li^+, K^+ and TBA^+ in DMF solvent; (d), (e) and (f) represent Li^+, K^+ and TBA^+ in THF solvent. The carbon atoms on TBA^+ are shown in green to distinguish them from the MOF carbon atoms.

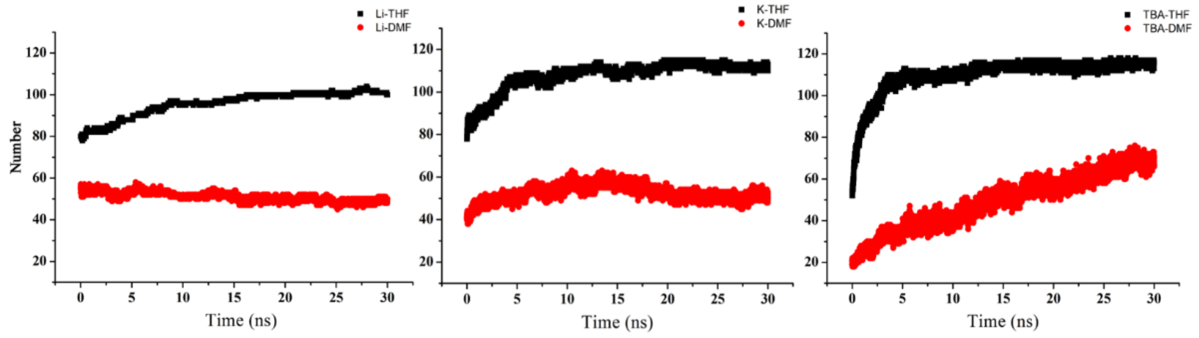

Figure S13. The number of cations within a given distance from the NDI oxygen atoms along the MD simulations in Zr(dcphOH-NDI). Only cations within 3 Å (for Li^+), 3.5 Å (K^+) and 4 Å (TBA^+) of O(NDI) are counted, respectively.

In THF solvent, the number of binding cations is much higher than that in DMF solvent, and both Li^+ and K^+ show stronger binding interactions with the linkers. Note that, although the experimental data for diffusion of K^+ in THF is not available, we simulated K^+ here for comparison. The radial distribution function (RDF) of cations among the NDI (**Figure S14**) clearly show that more Li^+ than K^+ are associated to the NDI in both DMF and THF solvents, and from the integral of RDF we see values that less than ¼ (there are four NDI oxygens per counter ion) indicating a distribution of bound and free ions. In THF, the ions are much more tightly bonded as indicated by the higher peak in the RDF of both K^+ and Li^+, and the integral which is approximately ¼ which corresponds to all cations bound.

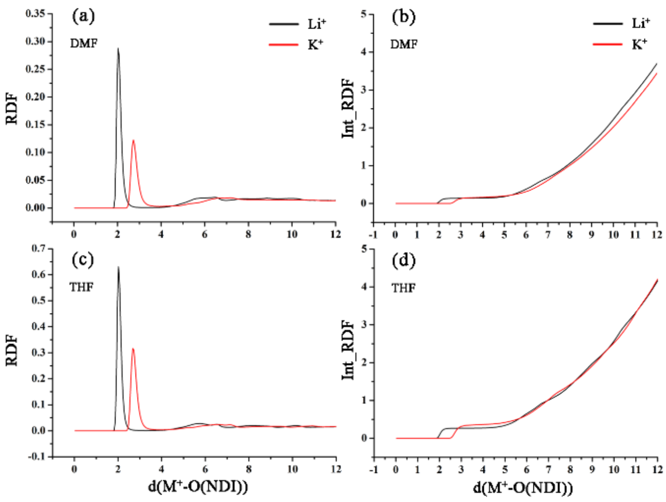

Figure S14. RDFs and integral of RDFs (Int_RDF) of Li^+ and K^+ around O(NDI) of the interconnected UiO-MOF in DMF (a & b) and THF (c & d) solvents.

To quantify the difference in the interaction free energy between the different cations to the NDI in the different solvents we performed the potential of mean force (PMF) calculations for the isolated linkers (but with the carboxylates replaced by hydrogen atoms). We observe a barrier for the dissociation of Li^+ and K^+ which is related to solvent rearrangement as the cation-O distance increases (**Figure S15**) The free energy barrier for dissociation of Li^+ in THF is about 8.0 kcal/mol whereas in DMF the value decreased to about 5.2 kcal/mol. For K^+, the free energy barrier is 5.0 kcal/mol in THF and 3.0 kcal/mol in DMF. TBA^+ shows a qualitatively different behavior and the dissociation free energy does change much as the solvent is changed.

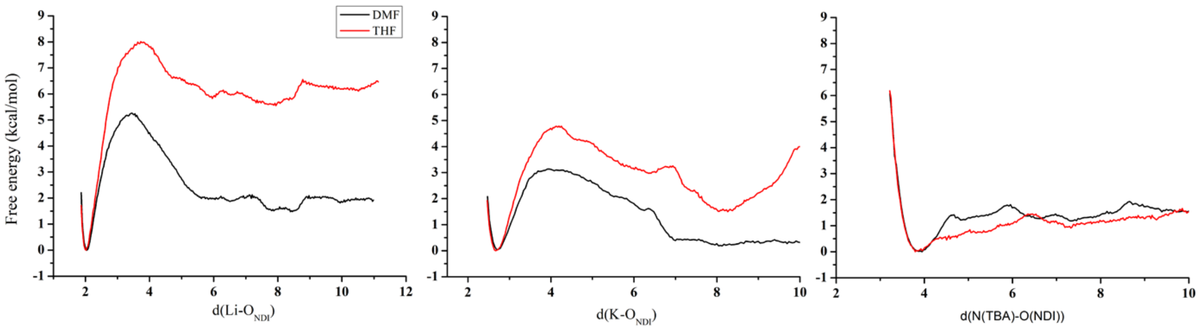

Figure S15. Potential mean force of cations binding with the reduced NDI. Note that the free energy increase for K^+ in THF solvent at distance longer than 8 Å was because the cation moves to another side of NDI and bonded with another NDI oxygen.

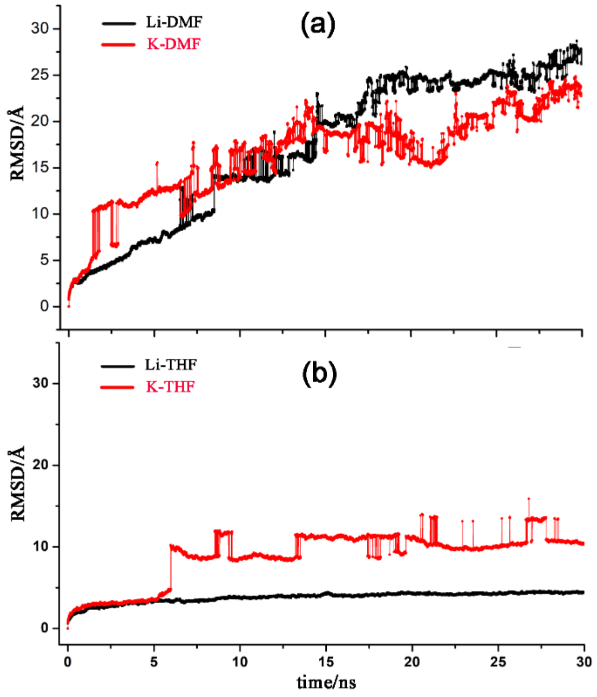

Figure S16. Root mean square deviation (RMSD) of the ion positions vs time in the simulations. (a): Li^+ and K^+ in DMF; (b): Li^+ and K^+ in THF. Sudden jumps are due to ions dissociating from MOF and entering the periodic box at other end. Main conclusion is that both ions move similarly, but much faster in DMF compared to THF.

DFT setup for single cluster with two linkers

One $Zr_6O_4(OH)_4$ cluster with 12 linkers was cut out from an MD trajectory. All except two linkers were replaced by acetate ions, and the ends of the two remaining linkers were neutralized with one proton each. The structures were fully optimized with the positions of the two terminal -COOH carbons restrained at the original position to simulate the constraints from the framework. The geometry optimizations were performed using the Jaguar 11.0 software package by Schrödinger[21] and the B3LYP-D3 functional[13, 22] and dispersion correction with the LACVP** basis set and core-potential for the metal atoms. The once reduced species without counterions was set up as an anionic species with a spin multiplicity of 2, and the geometry was optimized. The three ions were then placed at positions similar to the ones observed in the MD and the structure was optimized, giving a system neutral charge and multiplicity of 2. For the spin density calculations we included the PBF solvation model in Jaguar to account for solvent polarization. We used standard settings for dimethyl formamide. In **Figure 8** the spin densities of the Li^+ associated species is shown on the left, while the TBA+ associated species is shown on the right. Since charge delocalization can be exaggerated for some functionals we performed the calculation with the BHandHLYP functional, as well as the range separated ωb97x functional. Both gave results that were close to identical to B3LYP with the unpaired electron localized on one linker for the TBA^+ system and delocalized over two linkers for the Li^+ system.

6) References

1. Schneider, C. A.; Rasband, W. S.; Eliceiri, K. W., NIH Image to ImageJ: 25 years of image analysis. *Nat. Methods* **2012,** *9* (7), 671-675.
2. Johnson, B. A.; Bhunia, A.; Fei, H.; Cohen, S. M.; Ott, S., Development of a UiO-Type Thin Film Electrocatalysis Platform with Redox-Active Linkers. *J. Am. Chem. Soc.* **2018,** *140* (8), 2985-2994.
3. Bard, A. J.; Faulkner, L. R., *Electrochemical Methods: Fundamental and Applications*. 2 ed.; John Wiley & Sons, Inc.: Hoboken, N. J., 2001.
4. Cichocka, M. O.; Ångström, J.; Wang, B.; Zou, X.; Smeets, S., High-Throughput Continuous Rotation Electron Diffraction Data Acquisition via Software Automation. *J. Appl. Crystallogr.* **2018,** *51* (6), 1652-1661.
5. Kabsch, W., XDS. *Acta Crystallogr. Sect. D Biol. Crystallogr.* **2010,** *66* (2), 125-132.
6. Sheldrick, G. M., SHELXT - Integrated Space-Group and Crystal-Structure Determination. *Acta Crystallogr. Sect. A Found. Crystallogr.* **2015,** *71* (1), 3-8.
7. Sheldrick, G. M., A Short History of SHELX. *Acta Crystallogr. Sect. A Found. Crystallogr.* **2008,** *64* (1), 112-122.
8. Burla, M. C.; Caliandro, R.; Carrozzini, B.; Cascarano, G. L.; Cuocci, C.; Giacovazzo, C.; Mallamo, M.; Mazzone, A.; Polidori, G., Crystal Structure Determination and Refinement via SIR2014. *J. Appl. Crystallogr.* **2015,** *48* (1), 306-309.
9. Cavka, J. H.; Jakobsen, S.; Olsbye, U.; Guillou, N.; Lamberti, C.; Bordiga, S.; Lillerud, K. P., A New Zirconium Inorganic Building Brick Forming Metal Organic Frameworks with Exceptional Stability. *J. Am. Chem. Soc.* **2008,** *130*, 13850-13851.
10. Su, H.; Ahlquist, M. S. G., Nonbonded Zr4+ and Hf4+ Models for Simulations of Condensed Phase Metal–Organic Frameworks. *J. Phys. Chem. C* **2021,** *125* (11), 6471-6478.
11. Wang, J.; Wolf, R. M.; Caldwell, J. W.; Kollman, P. A.; Case, D. A., Development and Testing of a General Amber Force Field. *J. Comput. Chem.* **2004,** *25*, 1157-1174.
12. Wang, J.; Cieplak, P.; Kollman, P. A. How Well Does a Restrained Electrostatic Potential (RESP) Model Perform in Calculating Conformational Energies of Organic and Biological Molecules? J. Comput. Chem. 2000, 21, 1049−1074.
13. Becke, A.D. Density functional Thermochemistry. III. The Role of Exact Exchange. *J. Chem. Phys.* **1993**, 98, 5648–5652.
14. Barone, V., Cossi, M. Quantum calculation of molecular energies and energy gradients in solution by a conductor solvent model. *J. Phys. Chem. A* **1998**, 102, 1995-2001.
15. Case, D. A. B., R. M.; Cerutti, D. S.; Cheatham, T. E., III; Darden, T. A.; Duke, R. E.; Giese, T. J.; Gohlke, H.; Goetz, A. W.; Homeyer, N.; Izadi, S.; Janowski, P.; Kaus, J.; Kovalensko, A.; Lee, T. S.; LeGrand, S.; Li, P.; Lin, C.; Luchko, T.; Luo, R.; Madej, B.; Mermelstein, D.; Merz, K. M.; Monard, G.; Nguyen, H.; Nguyen, H. T.; Omelyan, I.; Onufriev, A.; Roe, D. R.; Roitberg, A.; Sagui, C.; Simmerling, C. L.; Botello-Smith, W. M.; Swails, J.; Walker, R. C.; Wang, J.; Wolf, R. M.; Wu, X.; Xiao, L.; Kollman, P. A., AMBER 2016, University of California: San Francisco. **2016**.
16. Izaguirre, J. A.; Catarello, D. P.; Wozniak, J. M.; Skeel, R. D. Langevin stabilization of molecular dynamics. J. Chem. Phys. 2001, 114, 2090−2098.
17. Berendsen, H. J. C.; Postma, J. P. M.; Vangunsteren, W. F.; Dinola, A.; Haak, J. R. Molecular-Dynamics with Coupling to an External Bath. J. Chem. Phys. 1984, 81, 3684−3690.
18. Souaille, M.; Roux, B., Extension to the weighted histogram analysis method: combining umbrella sampling with free energy calculations. *Comput. Phys. Commun.* **2001,** *135*, 40-57.

19. Grossman, A., ‘‘WHAM: the weighted histogram analysis method’’, version 2.0.6, http://membrane.urmc.rochester.edu/content/wham.

20. Kumar, S.; Rosenberg, J. M.; Bouzida, D.; Swendsen, R. H.; Kollman, P. A., THE weighted histogram analysis method for free-energy calculations on biomolecules. I. The method. *J. Comput. Chem.* **1992,** *13*, 1011-1021.

21. Jaguar, version 8.3, Schrodinger, Inc., New York, NY, 2014. Bochevarov, A. D.; Harder, E.; Hughes, T. F.; Greenwood, J. R.; Braden, D. A.; Philipp, D. M.; Friesner, R. A. Jaguar: A HighPerformance Quantum Chemistry Software Program with Strengths in Life and Materials Sciences. Int. J. Quantum Chem. 2013, 113, 2110-2142.

22. Grimme, S.; Antony, J.; Ehrlich, S.; Krieg, H. A Consistent and Accurate Ab Initio Parametrization of Density Functional Dispersion Correction (DFT-D) for the 94 Elements H−Pu. *J. Chem. Phys.* **2010**, 132, 154104.

7) **Mol2 File: Reduced NDI**

Mol2 file of the neutral NDI (named NDO) and reduced NDI (named NDR in the mol2 file) used in our MD simulations. Includes coordinates and partial atomic charges (last number in each row).

```
@<TRIPOS>MOLECULE
NDO
  52  57   1   0   0
SMALL
resp


@<TRIPOS>ATOM
   1 C1     -2.8990   -1.2490    0.1290 c      1 NDO     0.330925
   2 C2     -1.4090   -1.2300    0.0630 ca     1 NDO    -0.015300
   3 C3     -0.7110    0.0000    0.0320 ca     1 NDO    -0.004496
   4 C4     -1.4090    1.2300    0.0630 ca     1 NDO    -0.015300
   5 C5     -2.8990    1.2490    0.1290 c      1 NDO     0.330925
   6 C6     -0.7030   -2.4220    0.0310 ca     1 NDO    -0.085510
   7 C7      0.7110    0.0000   -0.0320 ca     1 NDO    -0.004496
   8 C8      1.4090   -1.2300   -0.0630 ca     1 NDO    -0.015300
   9 C9      0.7030   -2.4220   -0.0320 ca     1 NDO    -0.085510
  10 C10     2.8990   -1.2490   -0.1290 c      1 NDO     0.330925
  11 C11     2.8990    1.2490   -0.1290 c      1 NDO     0.330925
  12 C12     1.4090    1.2300   -0.0630 ca     1 NDO    -0.015300
  13 C13     0.7030    2.4220   -0.0310 ca     1 NDO    -0.085510
  14 C14    -0.7030    2.4220    0.0310 ca     1 NDO    -0.085510
  15 H15    -1.2640    3.3500    0.0560 ha     1 NDO     0.113220
  16 H16     1.2640    3.3500   -0.0560 ha     1 NDO     0.113220
  17 H17    -1.2640   -3.3500    0.0560 ha     1 NDO     0.113220
  18 H18     1.2640   -3.3500   -0.0560 ha     1 NDO     0.113220
  19 C19     4.9970    0.0000   -0.1900 ca     1 NDO     0.002517
  20 C20     5.7080    0.0000   -1.3910 ca     1 NDO    -0.179470
```

```
21 C21 5.6880 -0.0000 1.0270 ca 1 NDO 0.177287
22 C22 7.1000 0.0000 -1.3820 ca 1 NDO -0.133935
23 C23 7.0810 -0.0000 1.0250 ca 1 NDO -0.188996
24 C24 7.8020 0.0000 -0.1700 ca 1 NDO -0.003201
25 H25 7.6850 0.0000 -2.2960 ha 1 NDO 0.105329
26 H26 7.6620 -0.0000 1.9470 ha 1 NDO 0.096492
27 C27 -4.9970 0.0000 0.1900 ca 1 NDO 0.002517
28 C28 -5.7080 0.0000 1.3910 ca 1 NDO -0.179470
29 C29 -5.6880 -0.0000 -1.0260 ca 1 NDO 0.177287
30 C30 -7.1000 0.0000 1.3820 ca 1 NDO -0.133935
31 C31 -7.0820 -0.0000 -1.0250 ca 1 NDO -0.188996
32 C32 -7.8020 -0.0000 0.1700 ca 1 NDO -0.003201
33 H33 -7.6850 0.0000 2.2960 ha 1 NDO 0.105329
34 H34 -7.6620 -0.0000 -1.9470 ha 1 NDO 0.096492
35 C35 9.3590 -0.0000 -0.1360 c 1 NDO 0.629559
36 O36 9.8610 -0.0000 1.0170 o 1 NDO -0.679422
37 O37 9.9140 0.0000 -1.2610 o 1 NDO -0.679422
38 C38 -9.3590 -0.0000 0.1360 c 1 NDO 0.629559
39 O39 -9.8610 -0.0000 -1.0170 o 1 NDO -0.679422
40 O40 -9.9140 0.0000 1.2620 o 1 NDO -0.679422
41 O41 3.5230 -2.2960 -0.1510 o 1 NDO -0.411048
42 O42 3.5230 2.2960 -0.1510 o 1 NDO -0.411048
43 O43 -3.5230 -2.2960 0.1510 o 1 NDO -0.411048
44 O44 -3.5230 2.2960 0.1510 o 1 NDO -0.411048
45 N45 3.5480 0.0000 -0.1780 n 1 NDO -0.004271
46 N46 -3.5480 0.0000 0.1780 n 1 NDO -0.004271
47 H47 -5.1500 0.0000 2.3250 ha 1 NDO 0.111100
48 H48 5.1500 0.0000 -2.3250 ha 1 NDO 0.111100
49 O49 4.9460 -0.0000 2.1870 oh 1 NDO -0.500917
50 H50 5.5750 -0.0010 2.9200 ho 1 NDO 0.387272
51 O51 -4.9460 -0.0000 -2.1870 oh 1 NDO -0.500917
52 H52 -5.5760 -0.0000 -2.9200 ho 1 NDO 0.387272
@<TRIPOS>BOND
```

1	1	2	1
2	1	43	2
3	1	46	1
4	2	3	ar
5	2	6	ar
6	3	4	ar
7	3	7	ar
8	4	5	1
9	4	14	ar
10	5	44	2
11	5	46	1
12	6	9	ar
13	6	17	1
14	7	8	ar
15	7	12	ar
16	8	9	ar
17	8	10	1
18	9	18	1
19	10	41	2
20	10	45	1
21	11	12	1
22	11	42	2
23	11	45	1
24	12	13	ar
25	13	14	ar
26	13	16	1
27	14	15	1
28	19	20	ar
29	19	21	ar
30	19	45	1
31	20	22	ar
32	20	48	1
33	21	23	ar

```
  34   21   49 1
  35   22   24 ar
  36   22   25 1
  37   23   24 ar
  38   23   26 1
  39   24   35 1
  40   27   28 ar
  41   27   29 ar
  42   27   46 1
  43   28   30 ar
  44   28   47 1
  45   29   31 ar
  46   29   51 1
  47   30   32 ar
  48   30   33 1
  49   31   32 ar
  50   31   34 1
  51   32   38 1
  52   35   36 1
  53   35   37 1
  54   38   39 1
  55   38   40 1
  56   49   50 1
  57   51   52 1
@<TRIPOS>SUBSTRUCTURE
     1 NDO        1 TEMP              0 ****  ****    0 ROOT

@<TRIPOS>MOLECULE
NDR
  52   57    1    0    0
SMALL
resp
```

```
@<TRIPOS>ATOM
1 C1 2.8790 -1.2600 0.0810 c 1 NDR 0.289685
2 C2 1.4150 -1.2340 0.0390 ca 1 NDR -0.034657
3 C3 0.7190 -0.0000 0.0200 ca 1 NDR -0.011072
4 C4 1.4150 1.2340 0.0380 ca 1 NDR -0.034657
5 C5 2.8790 1.2600 0.0800 c 1 NDR 0.289685
6 C6 0.6920 -2.4470 0.0200 ca 1 NDR -0.140350
7 C7 -0.7190 -0.0000 -0.0200 ca 1 NDR -0.011072
8 C8 -1.4150 -1.2350 -0.0380 ca 1 NDR -0.034657
9 C9 -0.6910 -2.4470 -0.0180 ca 1 NDR -0.140350
10 C10 -2.8790 -1.2600 -0.0800 c 1 NDR 0.289685
11 C11 -2.8790 1.2600 -0.0810 c 1 NDR 0.289685
12 C12 -1.4150 1.2340 -0.0390 ca 1 NDR -0.034657
13 C13 -0.6920 2.4470 -0.0200 ca 1 NDR -0.140350
14 C14 0.6910 2.4470 0.0170 ca 1 NDR -0.140350
15 H15 1.2590 3.3710 0.0320 ha 1 NDR 0.089229
16 H16 -1.2600 3.3710 -0.0350 ha 1 NDR 0.089229
17 H17 1.2600 -3.3710 0.0350 ha 1 NDR 0.089229
18 H18 -1.2590 -3.3710 -0.0320 ha 1 NDR 0.089229
19 C19 -4.9710 -0.0000 -0.1430 ca 1 NDR 0.025111
20 C21 -5.7040 0.0010 1.0510 ca 1 NDR 0.191950
21 C20 -5.6680 -0.0010 -1.3530 ca 1 NDR -0.152831
22 C23 -7.0990 0.0010 1.0160 ca 1 NDR -0.215649
23 C22 -7.0630 -0.0010 -1.3830 ca 1 NDR -0.168381
24 C24 -7.7990 -0.0000 -0.1920 ca 1 NDR -0.002452
25 H26 -7.6920 0.0020 1.9300 ha 1 NDR 0.087722
26 H25 -7.6180 -0.0020 -2.3150 ha 1 NDR 0.095491
27 C27 4.9710 0.0000 0.1430 ca 1 NDR 0.025111
28 C29 5.7040 -0.0010 -1.0510 ca 1 NDR 0.191950
29 C28 5.6680 0.0010 1.3530 ca 1 NDR -0.152831
30 C31 7.0990 -0.0010 -1.0160 ca 1 NDR -0.215649
31 C30 7.0630 0.0010 1.3830 ca 1 NDR -0.168381
32 C32 7.7990 0.0000 0.1920 ca 1 NDR -0.002452
```

```
    33 H34        7.6920   -0.0010   -1.9300 ha      1 NDR     0.087722
    34 H33        7.6180    0.0020    2.3150 ha      1 NDR     0.095491
    35 C35       -9.3540   -0.0000   -0.1940 c       1 NDR     0.634244
    36 O36       -9.8950   -0.0010   -1.3300 o       1 NDR    -0.700741
    37 O37       -9.8950    0.0010    0.9440 o       1 NDR    -0.700741
    38 C38        9.3540    0.0000    0.1940 c       1 NDR     0.634244
    39 O39        9.8950    0.0010    1.3300 o       1 NDR    -0.700741
    40 O40        9.8950   -0.0010   -0.9440 o       1 NDR    -0.700741
    41 O41       -3.5400   -2.3020   -0.0970 o       1 NDR    -0.477622
    42 O42       -3.5400    2.3020   -0.0990 o       1 NDR    -0.477622
    43 O43        3.5400   -2.3020    0.0990 o       1 NDR    -0.477622
    44 O44        3.5400    2.3020    0.0970 o       1 NDR    -0.477622
    45 N45       -3.5270   -0.0000   -0.1150 n       1 NDR    -0.020140
    46 N46        3.5270    0.0000    0.1150 n       1 NDR    -0.020140
    47 H47        5.0890    0.0020    2.2730 ha      1 NDR     0.105263
    48 H48       -5.0890   -0.0020   -2.2730 ha      1 NDR     0.105263
    49 O49       -5.0100    0.0020    2.2410 oh      1 NDR    -0.490066
    50 H50       -5.6740    0.0030    2.9420 ho      1 NDR     0.369722
    51 O51        5.0100   -0.0020   -2.2410 oh      1 NDR    -0.490066
    52 H52        5.6740   -0.0020   -2.9420 ho      1 NDR     0.369722
@<TRIPOS>BOND
     1     1     2 1
     2     1    43 2
     3     1    46 1
     4     2     3 ar
     5     2     6 ar
     6     3     4 ar
     7     3     7 ar
     8     4     5 1
     9     4    14 ar
    10     5    44 2
    11     5    46 1
    12     6     9 ar
```

13	6	17	1
14	7	8	ar
15	7	12	ar
16	8	9	ar
17	8	10	1
18	9	18	1
19	10	41	2
20	10	45	1
21	11	12	1
22	11	42	2
23	11	45	1
24	12	13	ar
25	13	14	ar
26	13	16	1
27	14	15	1
28	19	20	ar
29	19	21	ar
30	19	45	1
31	20	22	ar
32	20	49	1
33	21	23	ar
34	21	48	1
35	22	24	ar
36	22	25	1
37	23	24	ar
38	23	26	1
39	24	35	1
40	27	28	ar
41	27	29	ar
42	27	46	1
43	28	30	ar
44	28	51	1
45	29	31	ar

```
46   29   47 1
47   30   32 ar
48   30   33 1
49   31   32 ar
50   31   34 1
51   32   38 1
52   35   36 1
53   35   37 1
54   38   39 1
55   38   40 1
56   49   50 1
57   51   52 1
@<TRIPOS>SUBSTRUCTURE
  1 NDR        1 TEMP            0 ****  ****    0 ROOT
```
